# High-quality de novo assembly of the *Eucommia ulmoides* haploid genome provides new insights into evolution and rubber biosynthesis

**DOI:** 10.1038/s41438-020-00406-w

**Published:** 2020-11-01

**Authors:** Yun Li, Hairong Wei, Jun Yang, Kang Du, Jiang Li, Ying Zhang, Tong Qiu, Zhao Liu, Yongyu Ren, Lianjun Song, Xiangyang Kang

**Affiliations:** 1 Beijing Advanced Innovation Center for Tree Breeding by Molecular Design, Beijing Forestry University, 100083 Beijing, People’s Republic of China; 2 National Engineering Laboratory for Tree Breeding, Beijing Forestry University, 100083 Beijing, People’s Republic of China; 3 College of Biological Sciences and Technology, Beijing Forestry University, 100083 Beijing, People’s Republic of China; 4 School of Forest Resources and Environmental, Science, Michigan Technological University, Houghton, MI 49931 USA; 5 Hebei Huayang Fine Seeds and Seedlings Co., Ltd., 054700 Hebei, People’s Republic of China

**Keywords:** Transcriptomics, Genome evolution, Plant breeding, Pharmacogenomics

## Abstract

We report the acquisition of a high-quality haploid chromosome-scale genome assembly for the first time in a tree species, *Eucommia ulmoides*, which is known for its rubber biosynthesis and medicinal applications. The assembly was obtained by applying PacBio and Hi–C technologies to a haploid that we specifically generated. Compared to the initial genome release, this one has significantly improved assembly quality. The scaffold N50 (53.15 MB) increased 28-fold, and the repetitive sequence content (520 Mb) increased by 158.24 Mb, whereas the number of gaps decreased from 104,772 to 128. A total of 92.87% of the 26,001 predicted protein-coding genes identified with multiple strategies were anchored to the 17 chromosomes. A new whole-genome duplication event was superimposed on the earlier γ paleohexaploidization event, and the expansion of long terminal repeats contributed greatly to the evolution of the genome. The more primitive rubber biosynthesis of this species, as opposed to that in *Hevea brasiliensis*, relies on the methylerythritol-phosphate pathway rather than the mevalonate pathway to synthesize isoprenyl diphosphate, as the MEP pathway operates predominantly in *trans*-polyisoprene-containing leaves and central peels. Chlorogenic acid biosynthesis pathway enzymes were preferentially expressed in leaves rather than in bark. This assembly with higher sequence contiguity can foster not only studies on genome structure and evolution, gene mapping, epigenetic analysis and functional genomics but also efforts to improve *E. ulmoides* for industrial and medical uses through genetic engineering.

## Introduction

Polyisoprene, a polymer of isoprene (C5H8), is the primary chemical constituent of natural rubber. Based on the chemical structure of the two isoprene isomers, natural rubber can be classified into *cis*-polyisoprene (CPI) and *trans*-polyisoprene (TPI)^1^. Currently, the Brazilian rubber tree is the only commercial source of natural CPI^2,3^. This tropical tree species faces several challenges, however, including a narrow range of suitable habitats, densely planted areas for latex tapping, and threats from serious diseases such as blight and insect pests^4,5^. For these reasons, the shortage of natural rubber caused by an increasing global demand has led both governments and industrial enterprises to look for substitutes for this essential material to synthesize various rubber products.

*Eucommia ulmoides* is a tertiary relic perennial tree of high value. It is an important temperate economic tree species that is capable of producing not only wood but also valuable raw biomaterials for extracting rubber and the active ingredients of a traditional Chinese medicine called Duzhong^6,7^. Unlike the Brazilian rubber tree, *E. ulmoides* is a well-known tree species for producing *trans*-rubber, which has unique characteristics such as high hardness, a low thermal expansion/contraction coefficient, good insulation, and resistance to acid and alkaline conditions^8–10^. For this reason, it can be used to manufacture cable insulation and golf balls^11,12^. Moreover, since blended rubber of CPI and TPI has some augmented mechanical properties and a different thermal conductivity and diffusivity^1^ compared to those of rubber with CPI only, TPI has been made into *cis*-rubber by vulcanization to achieve improved elasticity, resilience, tensile strength, viscosity, hardness and weather resistance^13^.

As previously reported^6^, TPI is especially enriched in the leaves, bark, and peels of *E. ulmoides* trees. For this reason, *E. ulmoides* is considered an ideal alternative or supplementary tree species for producing the raw materials of natural rubber^14,15^. Moreover, *E. ulmoides* can grow in many countries located in the northern temperate zone, where Brazilian rubber trees cannot be domesticated. In addition to its value in producing rubber, *E. ulmoides* is an important source of some active ingredients in traditional Chinese medicine. Its leaves and bark are especially rich in chlorogenic acid (CGA), an antioxidant that is known to slow the release of glucose into the bloodstream after a meal^16^ and reduce blood pressure^17^. In addition, it contains aucubin, rutin, quercetin and other unknown ingredients that may have great value for biopharmaceuticals^18,19^. Therefore, a high-quality genome is indispensable for genetic improvements related to *E. ulmoides* rubber productivity and medicinal uses in addition to wood.

In 2018, a draft of the *E. ulmoides* genome (v1.0) was released^15^. Due to high heterozygosity (0.8–1%) and a large percentage of repetitive sequences (greater than 60%)^15,20^, genome assembly from diploid sequences can easily incorporate chromosomal fragments from two homologous chromosomes in alternation and increase errors at the connections of contigs/scaffolds, thereby resulting in inaccurate assemblies with poor adjacency and increased linkage and base-error rates. To improve the quality of the *E. ulmoides* genome sequence, a haploid *E. ulmoides* (2*n* = *x* = 17) individual was generated through parthenogenesis and used for genome sequencing. The genome (v2.0) was acquired by assembling PacBio long reads and anchoring scaffolds based on information from Hi–C interaction confirmation. Using this high-quality genome, additional transcriptomic analyses of different tissues were undertaken to investigate rubber and CGA biosynthesis pathways in *E. ulmoides*. The genome sequence and gene annotation that we generated from the haploid will be instrumental for all studies on genome structure and evolution, gene linkage and mapping, epigenetic and chromatin-based analyses, and functional genomics as well as genetic engineering of *E. ulmoides* for industrial and medical uses.

## Results

### Induction of haploidy by high-temperature exposure

**Fig. 1:**
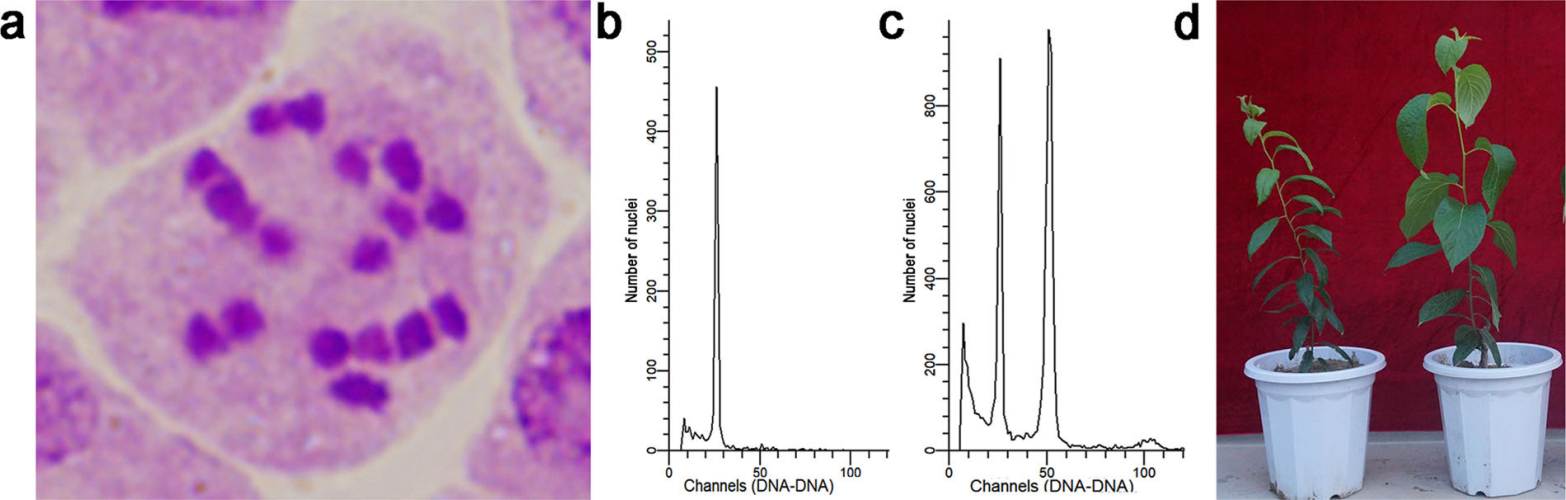
Karyotypic analysis of haploid plants generated through parthenogenesis under high-temperature treatments (48 °C for 6 h and 54 °C for 4 h) in *E. ulmoides*. **a** Somatic chromosome number of the haploids (2*n* = *x* = 17). **b** Ploidy levels obtained from 3-week-old first leaf samples from haploid plants by flow cytometric analysis. **c** Ploidy levels obtained from 3-week-old first leaf samples from a mixture of haploid and diploid plants by flow cytometric analysis. **d** A haploid plant (left) and diploid plant (right) of *E. ulmoides*

To induce haploidy through parthenogenesis, female flower buds of *E. ulmoides* in the developmental stage of embryo sac formation were treated with different combinations of high temperatures and treatment times, and untreated female flower buds were used as controls. A total of 2675 seeds were harvested after the treatments, and 835 plants were eventually obtained after sowing seeds and transplanting seedlings. Using flow cytometry and analysis of anatomical sections of stem tips, three authentic *Eucommia* haploid plants were obtained exclusively from the treated group (Fig. 1a–d). Of these, one haploid was obtained from continuous treatment at 48 °C for 6 h, while the other two haploid plants were obtained from continuous treatment at 54 °C for 4 h.

### Genome sequencing and assembly

We conducted de novo sequencing using the PacBio Sequel platform, which yielded a total of 97.26 Gb of raw data, representing a 95× coverage depth of the 1.02 Gb genome as estimated by 17-Kmer evaluation (Table S1 and Figs. S1 and S2). The PacBio long reads were corrected and assembled into 564 contigs using FALCON and Quiver^21^, with a contig N50 of 13.16 Mb (Table 1 and Table S2), which is 632 times longer than the contig N50 of the v1.0 genome. The total length of these contigs is 947.84 Mb of genome sequence (Tables S2 and S3), accounting for 92.93% of the estimated genome size. In addition, 133.74 Gb of high-quality Hi–C data with ~130× coverage was generated by the Illumina NovaSeq platform (Table S1). Valid high-quality Hi–C data were aligned with PacBio contigs using Burrows-Wheeler Aligner (BWA)^22^, and duplicates were removed with SAMTOOLS rmdup^23^ before LACHESIS^24^ was employed to assemble them into scaffolds based on linkage information and restriction enzyme sites. Finally, 145 long scaffolds were anchored to 17 linkage groups and further formed pseudochromosomes, accounting for 94.94% of the assembly (Table S4). The genome (v2.0) finally acquired contains a total of 501 scaffolds with a scaffold N50 of 53.15 Mb (Table 1 and Table S3), which is 28-fold longer than that in v1.0. Compared to the 104,772 gaps between adjacent scaffolds in v1.0, the number of gaps in v2.0 is only 128. Core Eukaryotic Genes Mapping Approach (CEGMA) evaluation^25^ was undertaken and showed that the newly assembled genome covered 230 (92.74%) of the 248 core eukaryotic genes (CEGs) completely and 8 (3.23%) of them partially, and the number of undetected CEGs was therefore 10 (4.04%) (Table S5). Genome completeness was assessed using Benchmarking Universal Single-Copy Orthologs (BUSCOs)^26^, which revealed complete genes (91.3%) and partial genes (1.7%) (Table S6). To further evaluate the assembly schemes, high-quality reads from a small-fragment library (350 bp) were aligned to the assembled genome. The results showed that the alignment rate was 99.18%, and the genome coverage was 99.86% (Table S7). A high degree of consistency between the Hi–C and PacBio results was also illustrated (Fig. S3). These results provide evidence that the new *E. ulmoides* genome assembled from the haploid is of high quality.

**Table 1:** Statistics for the *Eucommia* genome and gene annotation.

**Assembly**	
Estimate of genome size	1.02 Gb
Total assembly size	947.84 Mb
Number of contigs	564
N50 of contigs	13.16 Mb
Longest contigs	34.99 Mb
Sequence anchored to the Hi–C map	947.86 Mb
Number of scaffolds after Hi–C assembly	501
N50 of scaffolds after Hi–C assembly	53.15 Mb
Longest scaffold after Hi–C assembly	79.92 Mb
**Annotation**	
GC content	0.3517
Number of genes	26001
Percentage of gene length in genome	16.84%
Mean gene length	6138.21
Mean coding sequence length	1108.8
Mean exon number per gene	4.85
Mean exon length	228.56
Mean intron length	1305.93
rRNAs	2099
tRNAs	825
miRNAs	1032
snRNAs	875
Repeat content	62.50%

### Gene content and annotation

**Fig. 2:**
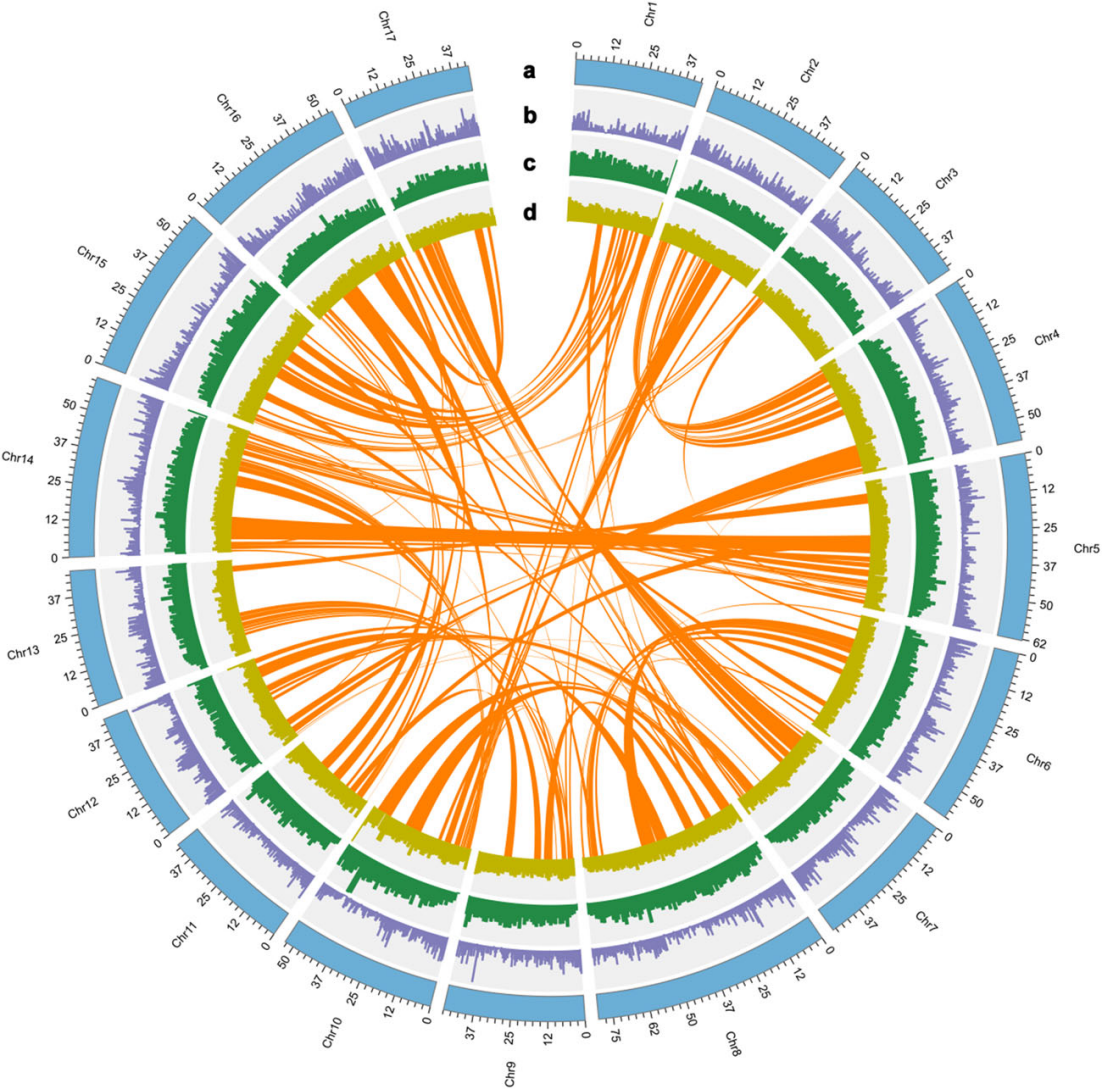
Overview of the *E. ulmoides* genome. **a** Lengths of pseudochromosomes (0.5 Mb window size (WS)); **b** gene density (0.3 Mb WS); **c** repeat density (1 Mb WS); **d** GC content (1 Mb WS). The colored lines in the center show links between syntenic blocks of at least five genes

Multiple strategies, including de novo prediction, homology-based methods, and transcript alignment, were employed for gene identification and annotation. Augustus^27^, GlimmerHMM^28^ and SNAP (http://homepage.mac.com/iankorf/) software were used for de novo gene prediction, whereas BLAST^29^ and Genewise^30^ were utilized to identify homologous open reading frames (Table S8). RNA-seq data derived from 12 different tissue types were assembled with Trinity^31^, and the assembled sequences were aligned against the *E. ulmoides* genome by PASA^32^. A total of 26,001 protein-coding genes, compared to 26,723 in v1.0, were predicted. The average length of the predicted genes was 6,138.21 bp, the average coding sequence (CDS) length was 1,108.80 bp, the average number of exons contained in each gene was 4.85, the average exon length was 228.56 bp, and the average intron length was 1,305.93 bp (Table S9 and Fig. S4). In total, 98.8% of the 26,001 predicted genes had homologs in at least one of the six functional protein databases aligned (Figs. S5, S6 and Table S10), and 24,148 genes could be localized to the chromosome, accounting for approximately 92.87% of the total predicted genes. The GC content, repeat density and gene density for each chromosome were plotted with Circos^33^ (Fig. 2). Many noncoding RNAs, including 2099 rRNAs, 825 tRNAs, 1032 miRNAs, and 875 snRNAs, were identified and are shown in Table S11.

**Fig. 3:**
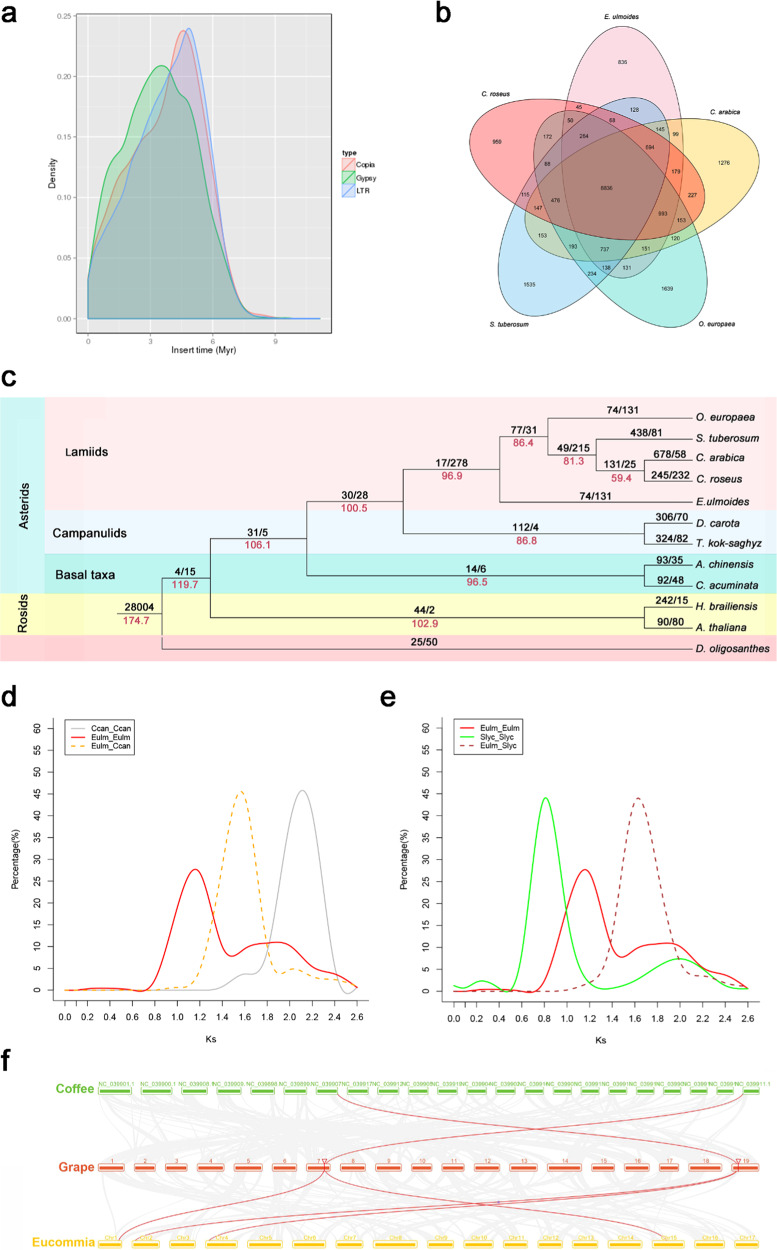
Evolution and synteny of the *E. ulmoides* genome. **a** The insertion times for intact LTR-RTs in the *E. ulmoides* genome. The insertion times for LTR-RTs were calculated by the formula *T* = *K*/2r. T insertion time; r synonymous mutations/site/Mya; K the divergence between the two LTRs. A substitution rate of 8.25 × 10^−9^ per site per year was used to calculate the insertion times. **b** Venn diagram of shared orthologs among the five species. Each number represents a gene family number. **c** Phylogenetic tree of 12 species based on orthologs of single-gene families. The number at the root (28,004) represents the number of gene families related to the common ancestor. The value above each branch denotes the number of gene families gained/lost during each round of genome duplication after diversification from the common ancestor. The red number below each branch denotes the speculated divergence time of each node. Bootstrap values for all nodes are above 50%. **d** Density distributions of Ks for paralogous genes. The peak values are shown in insets for *E. ulmoides* and *C. canephora*. **e** Density distributions of Ks for paralogous genes. The peak values are shown in insets for *E. ulmoides* and *S. lycopersicum*. **f** Schematic representation of syntenic genes among *E. ulmoides*, *V. vinifera* and *C. canephora*. Gray lines in the background indicate the collinear blocks of at least twenty genes within the *E. ulmoides* genome and other plant genomes, while the red lines highlight the syntenic gene pairs

The use of haploid *E. ulmoides* and PacBio data greatly facilitated the acquisition of repetitive sequences. A total of 592.43 Mb of repeat sequences, accounting for 62.50% of the genome length, were identified in the *E. ulmoides* v2.0 genome (Table 1 and Table S12). Combined homology-based and de novo approaches were used to identify transposable elements (TEs); long terminal repeats (LTRs), which are the main TE family in *E. ulmoides*, had a size of 520 Mb, accounting for 50.33% of the assembled genome. This was much longer than the 361.76 Mb of LTRs accounting for only 30.63% of the v1.0. genome. The copia- and gypsy-like LTRs covered 179.53 Mb and 267.69 Mb, respectively, accounting for 18.94% and 28.24% of the assembled genome. Only 3% of repeats in the v2.0 genome could not be classified into any known category (Table S13). These results indicate that the combination of a haploid genome and PacBio sequencing technology significantly improved the construction of repeat regions. Among the 8,264 intact LTR retrotransposons (LTR-RTs), 5254 and 2980 were gypsy-like and copia-like LTRs, respectively (Table S14). The estimated times for the intact LTR-RT insertion events indicate that the LTRs, copia-like LTRs and gypsy-like LTRs started to expand rapidly ~7.2 million years ago (Mya) and peaked ~5.2, 5.0 and 3.6 Mya, respectively. This time period falls within the Pliocene epoch (5.33–2.58 Mya) (Fig. 3a). The expansion of the copia type occurred before that of the gypsy type, causing intact LTR-RTs of the gypsy type to outnumber those of the copia type (Fig. 3a).

### Evolution of the *E. ulmoides* genome

Orthologous clustering of the predicted *E. ulmoides* proteins with those from 11 other representative species showed that 20,219 of the *E. ulmoides* protein-coding genes belong to 13,494 gene families with an average of 1.50 genes per family; 538 of these gene families are unique to *E. ulmoides* (Table S15 and Fig. S7)^34^. KEGG analysis revealed that *E. ulmoides*-specific genes are enriched in several pathways, including environmental adaptation, amino acid metabolism, glycan biosynthesis, and lipid metabolism (Table S16). Evolutionary analysis of gene families in *E. ulmoides*, together with those from 11 other plant species, was undertaken with CAFÉ (v 2.1)^35^. The results showed that 74 gene families expanded, while 131 gene families contracted (Fig. 3c). Comparative analysis of the protein-coding gene families of *E. ulmoides* and those in other closely related asterid species, including *Catharanthus roseus*, *Solanum tuberosum, Coffea arabica* and *Olea europaea*, revealed that 8836 of the 20,981 gene families were shared by these five species (Fig. 3b). The families that have expanded significantly are those that are mainly involved in cyanoamino acid metabolism, phenylpropionic acid biosynthesis, starch and sucrose metabolism, and glutathione metabolism (Table S17). This finding suggests that the expanded and unique gene families might be closely associated and have evolved to enhance environmental adaptability and to protect against herbivores and plant pathogens by increasing the production of high secondary metabolite contents.

There has been some controversy regarding classifying *E. ulmoides* into the lamiid lineage^15,36^. To determine its evolutionary status, a phylogenetic tree was constructed based on 133 single-copy gene families shared by *E. ulmoides* and 11 other species. In addition, their divergence time was estimated (Fig. 3c). The results indicate that *E. ulmoides* and four plant species from different categories of lamiids, including *C. roseus*, *S. tuberosum*, *C. arabica*, and *O. europaea*, are on the sister branches located in the basal group of the lamiid lineage. This result also supports the recent divergence of the campanulids *Daucus carota* and *Taraxacum kok-saghyz* and the recent divergence of Ericales (*Actinidia chinensis*) and Cornales (*Camptotheca acuminata*), which are sister groups located at the base of asterids^37^. The results of the phylogenetic tree are consistent with the systematic classification of the Angiosperm Phylogeny Group (APG IV, 2016). After calibrating the dates with those in the TimeTree database (http://www.timetree.org/), it is estimated that *E. ulmoides* diverged from campanulids ~100.5 Mya.

**Fig. 4:**
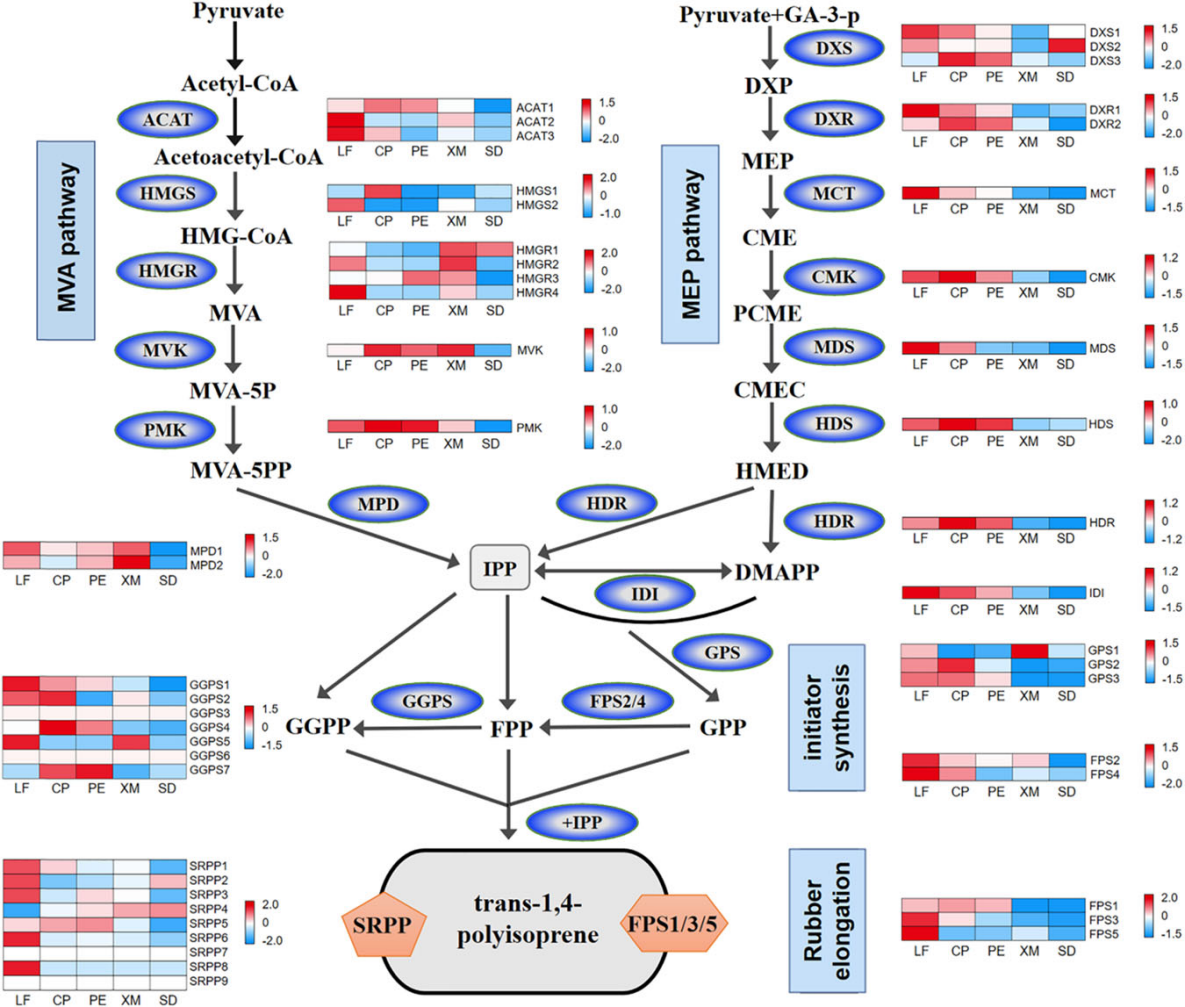
The *E. ulmoides* rubber biosynthesis pathway and expression profiles of genes involved in the pathway. The expression level is presented by log2-transformed fragments mapped per kilobase of transcript length per million total mapped reads (log2-FPKM). ACAT acetyl-coenzyme A (CoA) C-acetyltransferase; HMGS hydroxymethylglutaryl-CoA synthase; HMGR hydroxymethylglutaryl-CoA reductase; MVK mevalonate kinase; PMK 5-phosphomevalonate kinase; MPD mevalonate pyrophosphate decarboxylase; DXS 1-deoxy-d-xylulose 5-phosphate synthase; DXR 1-deoxy-d-xylulose 5-phosphate reductoisomerase; MCT 2-C-methyl-d-erythritol 4-phosphate cytidylyltransferase; CMK 4-(cytidine 5′-diphospho)-2-C-methyl-d-erythritol kinase; MDS 2-C-methyl-d-erythritol 2,4-cyclodiphosphate synthase; HDS 4-hydroxy-3-methylbut-2-enyl diphosphate synthase; HDR 4-hydroxy-3-methylbut-2-enyl diphosphate reductase; IDI isopentenyl diphosphate isomerase; GPS geranyl diphosphate synthase; FPS farnesyl diphosphate synthase; GGPS, geranylgeranyl diphosphate synthase; SRPP small rubber particle protein; Acetyl-CoA acetyl coenzyme-A; Acetoacetyl-CoA, 3-acetoacetyl-CoA; HMG-CoA, 3-hydroxy-3-methylglutaryl-CoA; MVA mevalonate; MVA-5P mevalonate-5-phosphate; MVA-5PP mevalonate-5-diphosphate; GA-3-P, glyceraldehyde 3-phosphate; DXP, 1-deoxy-d-xylulose 5-phosphate; MEP 2-C-methyl-d-erythritol 4-phosphate; CME 4-(cytidine 5′-diphospho)-2-C-methyl-d-erythritol; PCME 2-phospho-4-(cytidine 5′-diphospho)-2-C-methyl-d-erythritol; CMEC 2-C-methyl-d-erythritol 2,4-cyclodiphosphate; HMED 4-hydroxy-3-methylbut-2-enyl diphosphate; IPP isopentenyl diphosphate; DMAPP, dimethylallyl diphosphate; GPP geranyl diphosphate; FPP farnesyl diphosphate; GGPP, geranylgeranyl diphosphate. LF leaf; CP central peel; PE peel edge; XM xylem; SD seed

Whole-genome duplication (WGD) or polyploidization has occurred in the evolutionary history of most plant species^38^. The distribution of synonymous substitutions (Ks) and 4-fold synonymous third-codon transversions (4DTv) between duplicated genes was used to extrapolate WGD events. The results show that the *E. ulmoides* genome has two peaks in the Ks (Fig. 3d, e) and 4DTv distributions (Eulm_Eulm) (Fig. S8a, b), indicating that *E. ulmoides* may have undergone two WGD events. This is inconsistent with the results for the v1.0 genome^15^, where the peaks of γ duplication events in the Ks distribution analysis of *D. carota, Solanum lycopersicum*, and *Populus trichocarpa* were not conspicuous, presumably because less stringency was used in their analysis. Given that *Coffea canephora* and *Vitis vinifera* have only undergone an earlier γ paleohexaploidization event shared by all eudicots, the γ duplication events of *E. ulmoides* and *C. canephora* were very close to each other and resembled those in the *C. canephora* genome^39^. Collinearity analysis of *E. ulmoides*, *C. canephora* and *V. vinifera* with JCVI^40^ revealed a 2:1 synteny pattern between either *E. ulmoides* and *C. canephora* or *E. ulmoides* and *V. vinifera* (Figs. S9 and S10). QUOTA­ALIGN was then used to draw collinear dot plots to further validate the additional WGD upon γ paleohexaploidization (Figs. S11 and S12). As shown in Table S18, when the ratio of *E. ulmoides* to either coffee or grape was 1:1, *E. ulmoides* coverage was only 65.6% and 61.5% compared to 92.5% and 94%, respectively, in *C. canephora* and *V. vinifera*. When the ratios of *E. ulmoides* to *C. canephora* and *V. vinifera* were both 2:1, *E. ulmoides* coverage increased to 96.2% and 97%, respectively; thereafter, the coverage of *E. ulmoides* remained unchanged (<±0.5%), even when the ratio increased to a high value, such as 8:1. All of these results support that *E. ulmoides* underwent another WGD event after the earlier γ paleohexaploidization event shared by all eudicots (Fig. 3d–f and Table S18). Based on the repeated gene pairs in the genome, these two WGDs of *E. ulmoides* occurred 69.7 Mya and between 109.1 and 121.1 Mya, respectively (Table S19).

### Metabolic gene clusters and rubber biosynthesis pathways

Plant genes involved in secondary metabolic pathways are sometimes concentrated in specific genomic regions, resulting in biosynthesis gene clusters (BGCs)^35,41–43^. Using plantiSMASH^44^, we identified 26 BGCs that are associated with various secondary metabolic pathways in *E. ulmoides* (Table S20 and File S1). Among these 26 BGCs are 9 saccharide-related BGCs, 6 terpene-related BGCs, 4 alkaloid-related BGCs, 1 polyketide-alkaloid-related BGC, 1 saccharide-terpene-related BGC, and 1 lignin-polyketide-related BGC. The other four putative BGCs could not be assigned to a specific secondary metabolic pathway. The sizes of the BGCs identified vary from 32.01 to 2191.17 kb, which usually contain 3–5 core protein domains related to secondary metabolism. These tightly linked BCGs may facilitate cosegregation of coadaptive variation and limit unfit recombinant BCGs, resulting in more stable secondary metabolism.

*Trans*-polyisoprene (TPI) is known to be synthesized from isoprenyl diphosphate (IPP)^45,46^ through two pathways, namely, the mevalonate (MVA) pathway in the cytoplasm and the methylerythritol-phosphate (MEP) pathway in plastids^47,48^. A total of 47 candidate genes involved in the TPI biosynthesis pathway were identified in the genome (v2.0), including 13 genes involved in 6 reactions of the MVA pathway; 11 genes involved in 7 reactions of the MEP pathway; 12 genes involved in initial reactions for producing initiators or precursors, which include geranyl diphosphate synthases (GPSs), geranylgeranyl diphosphate synthases (GGPSs) and farnesyl diphosphate synthases (FPSs)); and 11 genes for ‘rubber elongation’ on TPI particles (Table S21 and Fig. S13).

Earlier studies showed that in addition to xylem and seeds, *E. ulmoides* leaves and peels contain a certain proportion of high-molecular-weight TPI^45^. The TPI content is rich in the central peels but relatively poor in the outer peel^49,50^. Differential expression analysis of genes in different tissues revealed that for the MEP pathway in *E. ulmoides*, at least one and sometimes two genes that encode enzymes to catalyze each step showed predominant expression in both TPI-containing leaves and central peels, indicating that the synthesis of IPP via the MEP pathway was in operation in both leaves and central peels. Although some MVA pathway genes were also expressed at relatively high levels, there was a lack of TPI-containing tissues in which all genes along the pathway were consistently “switched on”. Overall, MEP pathway genes had the highest or moderate to high expression levels, whereas MVA pathway genes had low to moderate expression levels (Table S21 and Fig. 4 and Fig. S1). It should be noted that prior to this study, we conducted a pilot experiment to examine the differential expression of pathway genes. Differential expression patterns in the pilot experiment were largely the same as those observed in the present study (see Table S21 and Fig. S14). These results suggest that IPP may be primarily and stably synthesized through MEP in the *E. ulmoides* trees used in our study.

All candidate genes involved in *trans*-IPP synthesis were obtained through homologous gene comparison, and the results are shown in Table S21. Three GPS, 7 GGPS and 4 FPS genes were identified in this *E. ulmoides* genome. Previous studies have shown that EuFPS homologs can be divided into two groups, namely, FPS 1/3/5 and FPS 2/4, which may play different roles in TPI synthesis^46^. FPS 1/3/5 homologs are involved in long-chain TPI biosynthesis, and FPS 2/4 homologs are in the same group as HbFPS1-3^15^. In the newly assembled genome, the evolutionary analysis also indicated that the FPS gene family can be divided into two groups (Figs. S15 and S16), and the expression of FPS1 is more than 200 times higher in leaves and peels with TPI than in xylem and seeds without TPI. This indicates that FPS1 may be a rate-limiting enzyme or at least an important enzyme in TPI synthesis.

As reported earlier, rubber elongation factor (REF)/small rubber particle protein (SRPP) family proteins are critically important for the biogenesis and stability of rubber particles^51,52^, and there was a positive correlation between REF1 expression and rubber yield in *H. brasiliensis*^53,54^. In the *E. ulmoides* genome (v2.0), as many as nine EuSRPP genes, which is fewer than the 12 *H. brasiliensis* SRPPs (HbSRPPs), were identified. However, no HbREF1 (138 amino acid) homologs were identified in *E. ulmoides*, indicating that there are differences in the mechanism of rubber biosynthesis between these two species. Based on the phylogenetic tree (Fig. S17), the SRPP family members of *E. ulmoides* are divided into two clades. The fragments mapped per kilobase of transcript length per million total mapped reads (FPKM) values of SRPP 1 and SRPP 5 in leaves and peels were significantly higher than 300, but there was no significant difference in the expression levels of SRPP5 between TPI tissue and non-TPI tissue. In contrast, the expression levels of SRPP 1 in TPI tissues (leaves and peels) were 7 times higher than those in non-TPI tissues (xylem and seeds). Presumably, SRPP 1 protein, similar to FPS1, may play a more important role in TPI synthesis. Based on the phylogenetic tree of REF/SRPP proteins from rubber-producing and non-rubber-producing plants^5,15,52,55^, REF/SRPP genes are scattered among some evolutionary branches of non-rubber-producing *O. europaea*, whereas most REF/SRPP genes in *T. kok-saghyz*, *H. brasiliensis* and *E. ulmoides* are distributed on one major branch (Fig. S18). This supports the rubber biosynthesis REF/SRPP gene families originating from multiple sources or from different evolutionary events, as demonstrated in previous studies^5,15,52^.

It is particularly noteworthy that genes within BGCs are involved in the synthesis of rubber initiators. Among the 6 predicted potential terpene secondary metabolite biosynthesis gene clusters, the core of Cluster 15 contains polyisoprene synthase. This gene cluster is 72.5 kb in size and is located at 8,851,940–9,257,031 bp on chromosome 4. The 16 genes in this gene cluster were functionally annotated (Table S22). Five core protein domains were predicted to be related to secondary metabolism by plantiSMASH^34^, including two encoding polyisoprene synthase (evm.model.Chr4.574 (GPS3) and evm.model.Chr4.575 (GGPS7)), one encoding cytochrome P450 (evm.model.Chr4.579) and two encoding dioxygenase (evm.model.Chr4.588 and evm.model.Chr4.593). In addition, nine members of the SRPP gene family were also present in two locally repeated gene clusters (BGCs): one was composed of three genes (evm.model.000124 f.13, evm.model.000124 f.14 and evm.model.000124 f.15), and the other was composed of four genes (evm.model.chr9.92, evm.model.chr9.94, evm.model.chr9.95, and evm.model.chr9.96), which originated from WGD events or tandem replication. The two remaining members are dispersed repeats on different chromosomes.

### Identification of genes involved in CGA synthesis

**Fig. 5:**
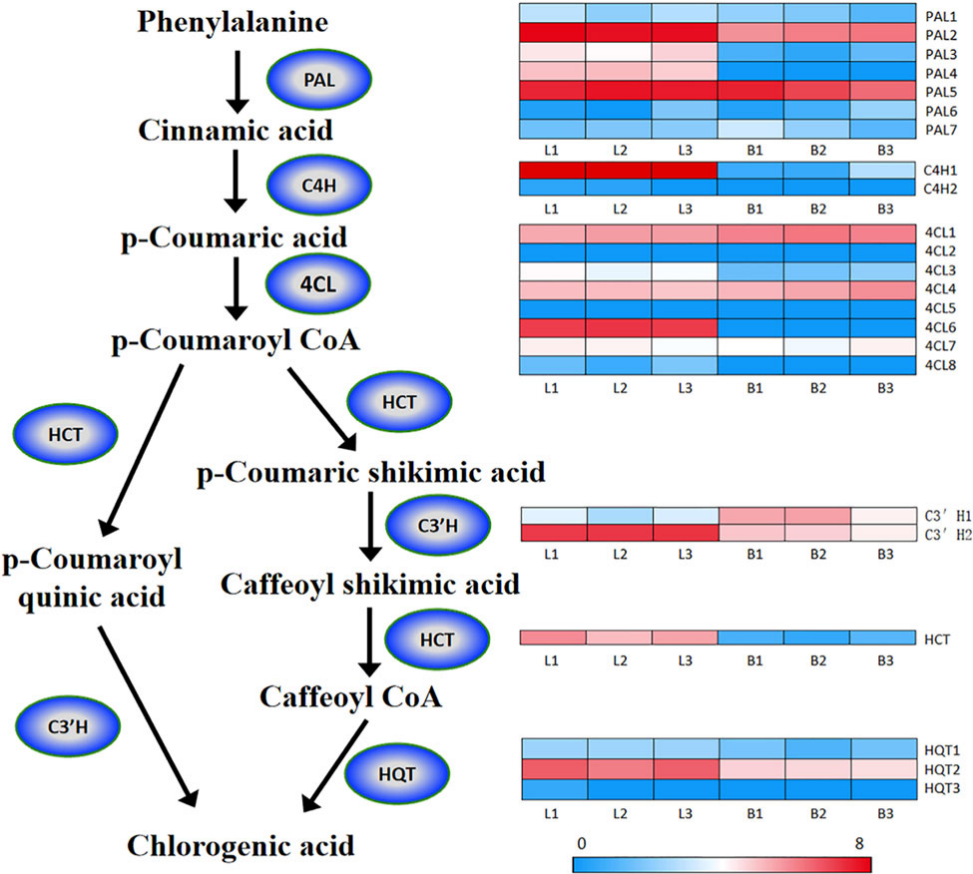
The chlorogenic acid biosynthesis pathway and expression profiles of genes involved in the pathway. The expression level is presented as log2-transformed fragments mapped per kilobase of transcript length per million total mapped reads (log2-FPKM). L1-L3 leaf; B1-3 bark. The enzymes involved are as follows: PAL phenylalanine ammonia-lyase; C4H cinnamate 4-hydroxylase; 4CL 4-coumaroyl-CoA ligase; HCT hydroxycinnamoyl-CoA: shikimate hydroxycinnamoyl transferase; C3′H *p*-coumaroyl ester 3′-hydroxylase; HQT hydroxycinnamoyl-CoA:quinate hydroxycinnamoyl transferase

As a traditional herbal medication, *E. ulmoides* accumulates CGA, the main phenolic compound, in its leaves and bark^56^. CGAs have long been known to be antioxidants that slow the release of glucose into the bloodstream after a meal^16^ and reduce blood pressure^17^. In addition, CGAs have antibacterial, antiviral and antitumor activities^57,58^. The biosynthesis of CGAs in *E. ulmoides* occurs through the phenylpropanoid pathway, where the initial reactions are catalyzed by phenylalanine ammonia lyase (PAL), cinnamate 4-hydroxylase (C4H), and 4-coumarin-CoA ligase (4CL) to generate *p*-coumarin-CoA (Fig. 5), which is a precursor for synthesizing CGAs through two different pathways. The first pathway produces *p*-coumaroyl quinic acid, which is then converted to CGAs. These two reactions are catalyzed by hydroxycinnamoyl-CoA shikimate/quinate hydroxycinnamoyl transferase (HCT) and *p*-coumaroyl ester 3′-hydroxylase (C3′H)^59^, respectively. The second pathway is *p*-coumarin-CoA → *p*-coumaric shikimic acid → caffeoyl shikimic acid → caffeoyl CoA → CGAs, and the four reactions are catalyzed by HCT, C3′H, HCT and hydroxycinnamoyl-CoA quinate HCT (HQT), respectively^60^. Through homologous gene comparison, 23 candidate genes related to 6 key enzyme gene families involved in the CGA biosynthesis pathway were identified in the *E. ulmoides* genome, including 7 PAL genes, 8 4CL genes, 2 C4H genes, 2 C3′H genes, 1 HCT gene, and 3 HQT genes (Fig. 5 and Table S23). Gene expression profile analysis showed that the expression levels of PAL (PAL2 and PAL5), C3′H2, C4H6, HQT2 and HCT were significantly higher than the expression levels of other genes. These genes may be key genes whose proteins catalyze CGA biosynthesis in *E. ulmoides*. In addition, some transcription factors (TFs) have also been reported to regulate CGA biosynthesis, including the *MYB*, *ZIP*, and *WRKY* families^61–64^. We identified 1,406 TF-encoding genes in *E. ulmoides*, representing 5.41% of the total genes. The annotation of these TFs is provided in Table S24. We specifically compiled the TFs that control biosynthesis of secondary metabolites (Table S25), which are composed primarily of phenolics, terpenoids, glucosinolates, and alkaloids and have been reported to serve the following roles: (1) protect plants against herbivores and pathogens^65,66^ and (2) constitute effective compounds in herbs in traditional medicine^67^. This information provides valuable information for further research on secondary metabolites in *E. ulmoides*.

## Discussion

Haploids play an important role in plant breeding and genome sequencing research. Typically, in vitro anther culture is the most widely used method to induce haploids^68^. However, anther culture cannot be achieved for the many plant species lacking an established tissue culture system. In this study, an in vivo branch bud heat treatment device^69^ was used for high-temperature treatment of *E. ulmoides* during the developmental stage of female embryo bud sacs, and three haploid lines were obtained. This shows that high-temperature treatment during embryo sac development is suitable and effective for inducing haploids in *E. ulmoides*. It also provides a reference for other plant species that do not have an established tissue culture system to induce apomixis at high temperature.

Various types of genetic and genomic research necessitate an accurate de novo whole-genome assembly as a foundation for developing genetic resources to understand evolution, genetics, inheritance, genomics, epigenetics and functional genomics. However, acquisition of a high-quality genome assembly is an arduous task, especially for species with high heterozygosity (or polymorphism) and/or highly repetitive sequences, including gene duplications, transposons, and short sequence repeats^70,71^. To circumvent these challenges, we specifically developed haploid *E. ulmoides* plants through parthenogenesis as described above. We used plant material from one of these haploids for genome sequencing, which can enhance the quality of genome assembly and the accuracy of gene annotation owing to the lack of heterozygosity. In addition, the use of advanced PacBio long reads and Hi–C data further enhances the quality of the genome. Compared to the initial genome released (v1.0)^15^, the current version (v2.0) has significantly improved assembly quality. For example, the scaffold number was reduced from 29,152 to 501, the scaffold N50 (53.15 MB) increased 28-fold compared to that of v1.0, and the number of gaps between scaffolds decreased from 104,772 in v1.0 to 128 in v2.0 (Table S26). A previous study in fish showed that the use of a haploid sequence for whole-genome assembly can increase the scaffold N50 by 3- to 10-fold^71^. We thus assume that the 28-fold gain in the scaffold N50 may also be ascribed to the other technologies we used, such as Hi–C. In addition, we obtained 520 Mb of repetitive sequence, which is 158.24 Mb longer than that of the v1.0 genome. Further evaluation with CEGMA revealed 238 (95.97%, complete + partial) and 230 (92.74%, complete) of 248 core eukaryotic genes in genome v2.0, in contrast with 232 (93.55%, complete + partial) and 196 (79.03%, complete) in genome v1.0 (Table S5). BUSCO analysis identified 93% of the 1,440 single-copy genes in genome v2.0, of which 91.3% were complete copies, which was higher than the 92.8% single-copy genes and 90.0% complete copies identified in genome v1.0 (Table S6). Although the 26,001 predicted genes in genome v2.0 is lower than the 26,723 predicted in genome v1.0, the results of the BUSCO and CEGMA evaluations indicate that the v2.0 genome annotation is more accurate and complete.

The *E. ulmoides* genome contains a high percentage of repetitive sequences, which poses a great challenge to genome assembly when short reads are used. This is because many short-read assemblers employ an algorithm called de-Bruijn graph representation, which connects short sequence fragments based on their overlapping subsequences. When the de-Bruijn graph is used to extend the assembly, the assembler must deal with repetitive sequences by finding the optimal path from multiple choices^71^. However, this is often impossible, especially when extending contigs through repetitive sequences longer than the read length, resulting in many fragmented assemblies that end in unresolved repetitive sequences^72^. Therefore, the use of haploid PacBio long reads can significantly reduce the complexity and enhance walk-throughs of repetitive sequences. The majority of repetitive sequences are present in the genome as TEs, which play important roles in genome reorganization and evolution because TE insertions can change genome structure and generate mutations from which new genes can be derived^73^. In addition, TEs can modify gene regulatory networks by dispersing vast numbers of promoter and enhancer transcription factor binding sites, insulator sequences, and repressive elements^74^. Given its important roles, a genome assembly without accurately assembled repetitive sequences will be deficient for studying genome evolution and gene regulation. The total length of LTRs in the v2.0 genome is ~520 Mb, which greatly exceeds the 361.76 Mb in genome v1.0. The v2.0 genome contains only 3% noncategorical repeats, which is far less than the 17.16% in the v1.0 genome. Further analysis shows that most LTR-RT expansions occurred in the Pliocene 5.2 Mya. From the perspective of fossil history, the distribution range of *E. ulmoides* was almost throughout the Northern Hemisphere during the Miocene, and it began to shrink in the Neogene^75^. The reason is that during the Pliocene, the temperature and atmospheric carbon dioxide concentration continued to decline, and in the Pleistocene, Earth entered the glacial period, which changed plant growth and distribution zones^76^. Changes in environmental pressures led to the activation of TEs and plant genome reorganization as well as gene expansion^77,78^. Half of the resistance gene analogs in this genome were derived from TE insertions (File S2), suggesting that TE burst might have played an important role in *E. ulmoides* evolution and stress response.

The evolutionary history of *E. ulmoides* is further confirmed to be different from that of genome v1.0^15^. According to the taxonomy of the Angiosperm Phylogeny Group (APG IV, 2016), *E. ulmoides* is a latex-producing deciduous species in Garryales, which belongs to lamiids. Wuyun *et al*.^15^ concluded that *E. ulmoides* is a sister taxon of lamiids and campanulids. In this study, a phylogenetic tree based on genome v2.0 showed that *E. ulmoides* belongs to lamiids, which is consistent with the systematic classification of angiosperms by The Angiosperm Phylogeny Group APG IV (2016). The inconsistency might arise from the improved genome (2.0) and the selection of more lamiid plants to build phylogenetic trees. WGD or polyploidization has played an important role in plant genome evolution^79^. A previous study on genome v1.0 concluded that there was no additional WGD upon the γ paleohexaploidization event^15^. However, the present study provides multiple lines of new evidence that the *E. ulmoides* genome was marked by a lineage-specific paleotetraploidization event superimposed on the earlier paleohexaploidization event shared by all eudicots. By comparing syntenic patterns between *E. ulmoides* and grape/coffee, we obtained a stable 2:1 synteny ratio (Fig. 3f and Table S18). These results provide a basis for future comparative genomics analyses of asterid evolution.

The IPP derived from the MVA and MEP pathways is considered a substrate for synthetic TPI^45,80^. ^13^C isotopic labeling showed that TPI was obtained from both the MVA and MEP pathways in *E. ulmoides*^45^. Studies in *H. brasiliensis* and dandelion-type plants have shown that the MVA pathway is mainly involved in the synthesis of *cis*-rubbers^5,52,80,81^. Previous research using the v1.0 genome showed that *Eucommia* is similar to *H. brasiliensis* and *T. kok-saghyz*, and the main route of IPP synthesis is the MVA pathway^15^. However, a different result was obtained in this study. At least one enzyme in each process of the MEP pathway in *E. ulmoides* was predominantly expressed in the TPI-containing leaves and central peel. Therefore, the MEP pathway, rather than the MVA pathway, plays a major role in the synthesis of IPP for TPI biosynthesis. Pilot studies conducted previously produced similar results (Table S21 and Fig. S14). Such a discrepancy from the results of Wuyun *et al* may result from variation in the tree population. It is possible that the trees we studied are from different provenances and have adapted to different ecotypes or climate types. This variation may provide an opportunity for selective breeding for the improvement of TPI synthesis.

To date, four protein families related to long-chain CPI rubber elongation have been characterized: *cis*-prenyltransferases (CPTs), SRPP, REF and Nogo-B receptor (NgBR)-like protein^51,53,82,83^. CPT is responsible for adding IPP to the substrate in the *cis*-configuration to form long-chain CPI^82,84^. REF/SRPP is involved in the stabilization of the rubber particle membrane^85,86^. NgBR-like protein is a homolog of the human Nogo-B receptor and has been demonstrated to be essential for rubber biosynthesis in dandelion^83^. The NgBR-like protein functions as a mediating protein between REF and CPT to form the ternary complex to increase CPT activity^87^. However, the details of long-chain TPI biosynthesis after IPP formation in *E. ulmoides* via the MVA and MEP pathways remain largely unknown. Wuyun *et al*.^15^ found that some key amino acids in the conserved features/sites of FPS 1, 3, and 5 differed from those of FPS 2 and 4 and all three *HbFPS1-3*. At the same time, long-chain TPI was obtained after overexpression of FPS5 in tobacco, indicating that FPS 1, 3, and 5 may participate in the synthesis of long-chain TPI. Wuyun *et al*.^15^ also found that the expression levels of EuSRPP1, 2, and 7 instead of REFs corresponded to the accumulation of Eu-rubber. The expression levels of three REF genes were almost all zero in expression in all tissues examined^15^. In our study, we failed to annotate FPS5 in the first round of gene discovery because of its low expression levels in most tissues (Table S21), indicating the possibility of increasing TPI synthesis by overexpressing FPS1. In the v2.0 genome, only 9 SRPP gene family members were detected, and no genes that are homologous to HbREF1 (138 amino acids) were identified, indicating that they may be pseudogenes with incomplete gene sequences arising from the fragmentation of the contig/scaffold sequences in the v1.0 genome. In addition, 3 CPT genes and an NgBR homologous gene were found in the v2.0 genome, and their expression levels were much lower than those of FPS1 and SRPP1 (Table S21). This suggests that although CPT, REF, and NgBR-like proteins were reported to participate in long-chain CPI biosynthesis, whether they are functionally involved in the biosynthesis of long-chain TPI is unclear and needs to be further investigated. Given that the rubber in *E. ulmoides* is pure TPI^6,88^, it is still not possible to sketch the biochemical scenario for long-chain TPI synthesis in *E. ulmoides* with only the genes whose counterparts have been reported in other species as being involved in CPI synthesis. Clearly, more functional genes and enzymes need to be identified and characterized to delineate the underlying biochemical reactions and pathways of TPI synthesis. Corresponding to CPT, there should be at least one *trans*-prenyltransferase (TPT) enzyme that can add IPP in a *trans* configuration during the synthesis of long-chain TPI in *E. ulmoides*. TPT is a large gene family. Its main function is to add IPP to the precursor in the *trans* configuration. According to the chain length of the product, it can be divided into short-chain and long-chain TPTs, where short-chain TPTs are widely found in plants, mainly including geranyl GPS, FPS, and GGPS^5,52,89,90^. However, the long-chain TPT responsible for adding IPP to precursors for long-chain TPI rubber elongation is still unknown. Therefore, in addition to identifying long-chain TPTs, the functions of FPS and SRPP in Eu-rubber synthesis also need to be further characterized because existing evidence indicates that they may play a role during the synthesis of long-chain TPI^15^. Such divergence among species implies that the biosynthesis of polyisoprene in angiosperms is polyphyletic in origin.

In conclusion, the haploid *E. ulmoides* genome sequencing project has provided a highly accurate and contiguous reference genome sequence for evolutionary and genomic studies. Our preliminary analyses have added valuable information to the genomic resources of lamiids and led to novel conclusions regarding its evolution in terms of WGD, BGCs and rubber synthesis pathways. We believe that this work will be instrumental not only for accelerating evolutionary, genetic and genomic studies in *E. ulmoides* but also for advancing our understanding of the molecular mechanisms underlying the biosynthesis and regulation of rubber and chlorogenic acid as well as other valuable secondary metabolites such as rutin and quercetin.

## Materials and methods

### Induction of haploid by high-temperature exposure

The female flower buds of *E. ulmoides* in the developmental stage of embryo sac formation were treated at either 48 °C or 54 °C for 4–6 h, and untreated female flower buds were used as controls. Seeds were harvested and planted in a room at 25 °C for germination. Karyotyping was conducted with the aid of flow cytometry and anatomical sections to identify authentic *Eucommia* haploid plants.

### Plant material

Fresh leaves of haploid *E. ulmoides* plants originally induced by parthenogenesis were grown in a greenhouse for genome sequencing and Hi–C analysis. Apical buds, young leaves, mature leaves, old leaves, bark, roots, peels, seeds, lateral buds, male flowers (SRX2447963, SRX2447934, SRX2447933, and SRX1160206), female flowers (SRX1160207), and stem tissue of *E. ulmoides* were harvested, immediately frozen in liquid nitrogen and stored at −80 °C for RNA-seq experiments. The resulting RNA-seq data were deposited into the Sequence Read Archive (SRA) (https://www.ncbi.nlm.nih.gov/sra) public repository.

### SMRT long-read sequencing

The DNeasy Plant Mini Kit (TIANGEN, Beijing, China) was used to extract high-quality genomic DNA from young leaves of haploid *E. ulmoides*. For PacBio sequencing, a single-molecule real-time (SMRT) bell library with a 40-kb insert fragment length was constructed according to the manufacturer’s protocol (PacBio, CA).

### Illumina short-read sequencing

For Illumina (San Diego, CA) sequencing, 350-bp libraries with an average insert length of 350 bp were prepared using the NEBNext Ultra DNA Library Prep Kit and sequenced using the Illumina HiSeq X Ten platform at the Novogene Bioinformatics Institute, Beijing.

### Hi–C library construction and sequencing

A Hi–C library was prepared using the Dovetail^TM^ Hi–C Library Preparation Kit. Nuclear chromatin was fixed with formaldehyde. Fixed chromatin was digested with DpnII, and the sticky ends were filled with biotinylated nucleotides at the digested DNA ends prior to ligation. The chromatin was then reverse-crosslinked to free DNA from proteins, and the purified DNA was processed to remove any free biotin from the linked fragments. The DNA was then cut into 350-bp fragments, and the biotinylated fragments were enriched by pulling down with *streptavidin*-coated magnetic *beads*, followed by PCR amplification to generate a library. The size of the inserted fragment was checked using a Qubit 2.0 fluorometer and an Agilent 2100 fluorometer. The effective concentrations of the libraries were accurately quantified by Q-PCR to confirm their quality, and the libraries were sequenced on the Illumina NovaSeq platform.

### Genome assembly

Before assembling, the ‘daligner’ option in the FALCON assembler was used to correct the errors in PacBio long reads using PacBio short reads less than 6000 bp in length to generate consensus sequences^91^. Then, FALCON was used to identify the overlaps between all pairs of preassembled error-corrected reads. The read overlaps were used to construct a directed string graph following Myers’ algorithm. Contigs were constructed by finding the paths from the string graph (falcon_sense_option = - output_multi -min_idt 0.70 -min_cov 4 -max_n_read 300 -n_core 12; overlap_filtering_setting = -max_diff 100 -max_cov 100 -min_cov 4 -n_core 12). Error correction of the preceding assembly was performed by the consensus–calling algorithm Quiver with PacBio reads^21^. The Illumina reads were also used to correct the contigs with Pilon^92^. The heterozygosity in the error-corrected contigs was then removed by purge_haplotigs with Illumina clean reads.

The Hi–C clean data were aligned to the preceding assembly using BWA software^22^. SAMTOOLS was used to remove duplicates (parameters: rmdup) and non-aligned data^23^, and only the read pairs with both reads in the pair aligned to contigs were considered for scaffolding. The physical coverage of each read pair was defined as the total bases spanned by the sequences of the reads and the gap between the two reads when mapped to contigs. The per base physical coverage of each base in the contig was defined as the number of read pairs that physically included it. Given this, misassembly was detected by a sudden drop in the per-base physical coverage in a contig. According to the physical coverage of the alignment result, misassemblies were sheared to correct errors by SALSA^93^. Based on the linkage information and restriction enzyme sites, the string graph formulation was used to construct the scaffold graph with LACHESIS^24^. Finally, the scaffolds were anchored to 17 chromosomes, and then the interaction matrix heat map of all chromosomes was visualized with a resolution of 500 kb.

### Evaluation of the genome assembly

To evaluate the accuracy and completeness of the assembled *Eucommia* genome, Burrows-Wheeler Aligner (BWA)^22^ software was used to compare a small-fragment library with an average insert length of 350 bp to the assembled genome. The alignment rate, extent of genome coverage, and depth distribution were used to assess assembly integrity and sequencing uniformity. CEGMA^25^ and BUSCO^26^ were used to assess the integrity of the final genome assembly.

Additional detailed methods, including annotation of repetitive sequences, gene prediction, phylogenetic tree reconstruction, species divergence time estimation, expansion and contraction of gene families, genome synteny and WGD assessment, transcriptome sequencing, and analysis, identification of resistance genes, identification of CGA synthesis-related genes, identification of transcription factors and phylogenetic analysis, are available in File S2.

## Supplementary information

File S1

Supplemental file 2

Figure S1-S19

Supplemental table

Table S16

Table S17

Table S20

Table S21

Table S22

Table S24

Table S25

## Data Availability

All sequencing reads and the genome assembly have been deposited in the NCBI database under accession number PRJNA599775.
