# Supplementary material for: High-quality de novo assembly of the Eucommia ulmoides haploid genome provides new insights into evolution and rubber biosynthesis
Source: Hortic Res. 2020 Nov 1;7:183. doi: 10.1038/s41438-020-00406-w (PMC7603500; doi:10.1038/s41438-020-00406-w)

1. 000108F - Gene cluster 1. Type = terpene. Location: 527345 - 953886 nt.


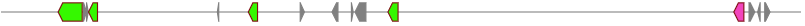


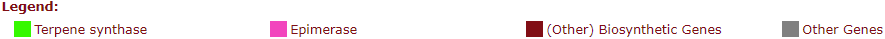


1. Chr1 - Gene cluster 2. Type = saccharide. Location: 24365301 - 24601286 nt.


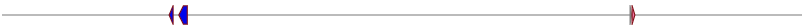


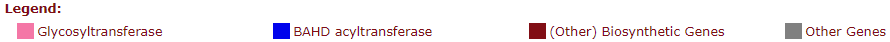


1. Chr10 - Gene cluster 3. Type = polyketide-alkaloid. Location: 34090282 - 34701586 nt.


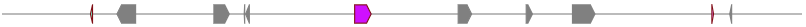


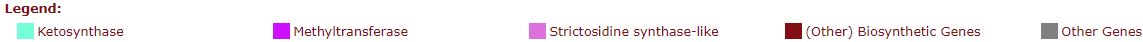


1. Chr12 - Gene cluster 4. Type = lignan-polyketide. Location: 24304852 - 24660228 nt.


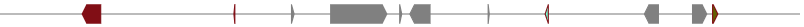


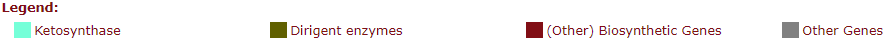


1. Chr13 - Gene cluster 5. Type = terpene. Location: 2095257 - 2127266 nt.


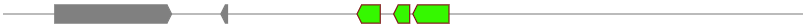


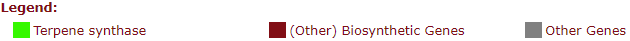


1. Chr13 - Gene cluster 6. Type = terpene. Location: 2844430 - 3127765 nt.


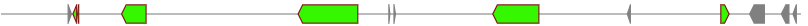


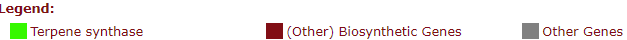


1. Chr13 - Gene cluster 7. Type = putative. Location: 45613843 - 45686426 nt.


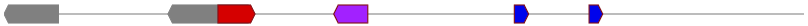


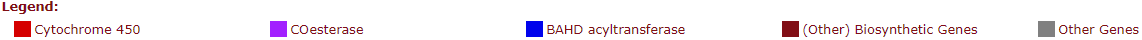


1. Chr14 - Gene cluster 8. Type = saccharide. Location: 49941764 - 50106808 nt.


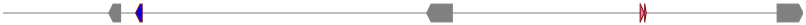


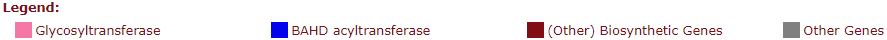


1. Chr14 - Gene cluster 9. Type = terpene. Location: 58773290 - 58885218 nt.


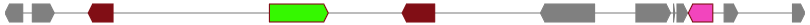


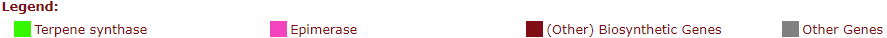


1. Chr15 - Gene cluster 10. Type = saccharide. Location: 19354995 - 21029795 nt.


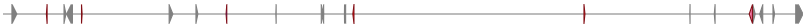


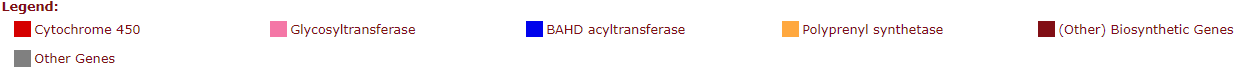


1. Chr15 - Gene cluster 11. Type = saccharide. Location: 51038570 - 51782116 nt.


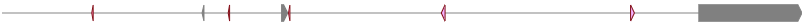


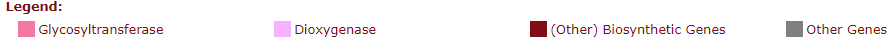


1. Chr16 - Gene cluster 12. Type = saccharide. Location: 1300810 - 1508277 nt.


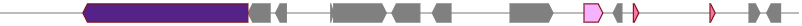


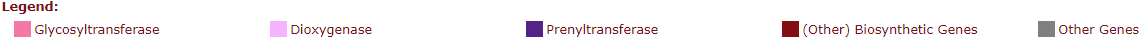


1. Chr16 - Gene cluster 13. Type = saccharide. Location: 48114831 - 48434710 nt.


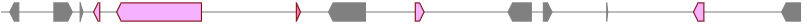


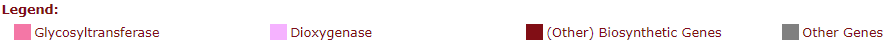


1. Chr3 - Gene cluster 14. Type = alkaloid. Location: 3850408 - 3965581 nt.


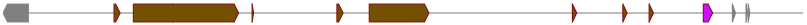


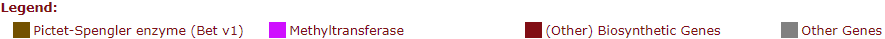


1. Chr4 - Gene cluster 15. Type = putative. Location: 8851940 - 9257031 nt.


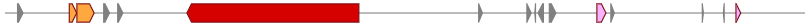


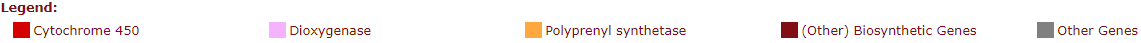


1. Chr4 - Gene cluster 16. Type = terpene. Location: 17000832 - 17208443 nt.


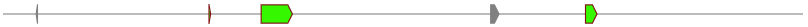


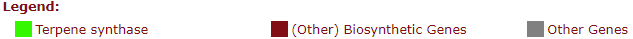


1. Chr4 - Gene cluster 17. Type = saccharide. Location: 28624930 - 29101928 nt.


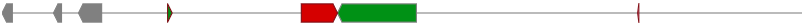


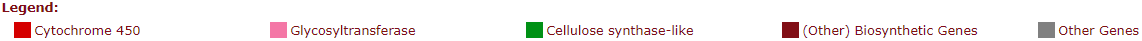


1. Chr6 - Gene cluster 18. Type = terpene. Location: 12860195 - 13281110 nt.


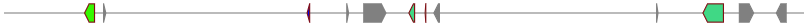


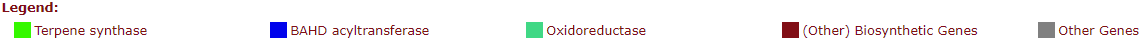


1. Chr7 - Gene cluster 19. Type = saccharide-terpene. Location: 3878083 - 4034217 nt.


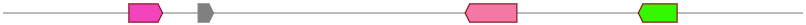


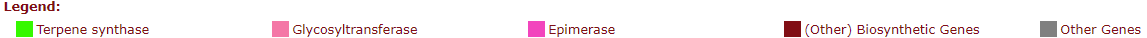


1. Chr7 - Gene cluster 20. Type = putative. Location: 45523106 - 45699272 nt.


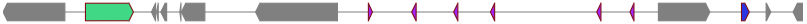


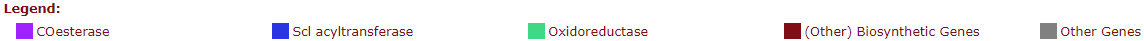


1. Chr8 - Gene cluster 21. Type = putative. Location: 1742698 - 2455313 nt.


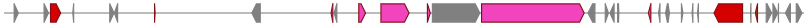


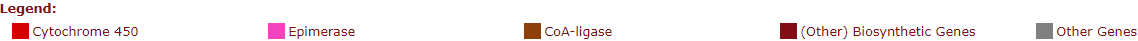


1. Chr8 - Gene cluster 22. Type = saccharide. Location: 7745380 - 7971419 nt.


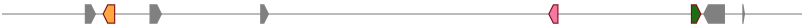


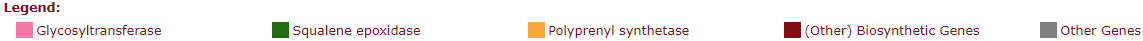


1. Chr8 - Gene Cluster 23. Type = saccharide. Location: 8106359 - 8405398 nt.


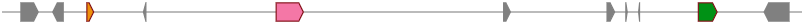


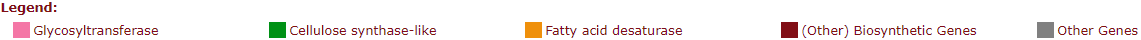


1. Chr8 - Gene cluster 24. Type = alkaloid. Location: 46436644 - 46674123 nt.


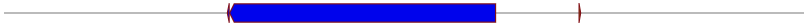


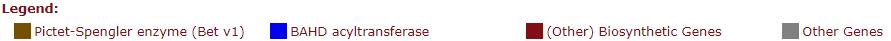


1. Chr8 - Gene cluster 25. Type = alkaloid. Location: 61933120 - 64124293 nt.


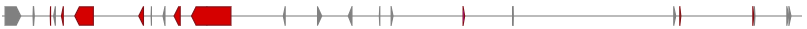


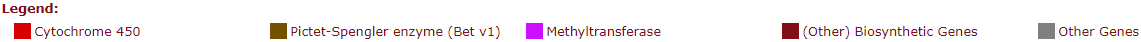


1. Chr8 - Gene cluster 26. Type = alkaloid. Location: 72061033 - 72257615 nt.


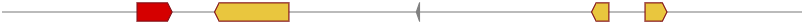


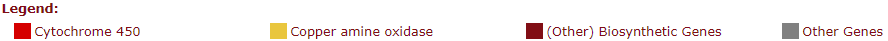

Supplement: Supplementary file 1 — File S1 [file 41438_2020_406_MOESM1_ESM.doc]
