# Supplementary material for: High-quality de novo assembly of the Eucommia ulmoides haploid genome provides new insights into evolution and rubber biosynthesis
Source: Hortic Res. 2020 Nov 1;7:183. doi: 10.1038/s41438-020-00406-w (PMC7603500; doi:10.1038/s41438-020-00406-w)
Supplement: Supplementary file 2 — Supplemental file 2 [file 41438_2020_406_MOESM2_ESM.doc]

***Identification of genes involved in pest and disease resistance***

Most of the proteins encoded by resistance gene analogues (RGAs) contain conserved domains, and the four major families of RGAs include: nucleotide binding site (NBS) domain proteins, transmembrane-coiled-coil (TM-CC) proteins, receptor like proteins (RLPs) and receptor-like kinases (RLKs). RLPs and RLKs are membrane-bound signalling molecules with an extracellular receptor domain^41^. Using a genome-wide scanning pipeline^1^, we identified 761 RGAs in *E. ulmoides* (Table S27). A total of 441 (58%) RGAs in *E. ulmoides* are of RLK family, and there were 139 (18%) NBS-related RGAs. We noticed that the majority of RGAs clustered near two ends of chromosomes (Figure S12). Unlike RGA genes in *D. carota* which are arranged in tandem replication, most RGA genes in *E. ulmoides* are in dispersed repeats (TE insertions) (Table S28), which may reflect the experience of different evolutionary scenarios. There are two RGAs, one RLK-encoded RGA and one NBS protein, which are among the 89 genes that were identified as the intact LTR-RTs. These two genes can be assigned to known resistance genes using the reference pathogenesis-related (PR) proteins in the latest version of the Plant Resistance Gene database (PRGdb)^2^. These results suggest that the environmental changes driven expansion of LTRs played a role in *E. ulmoides* genome evolution, which impacted RGA family.

**Methods**

***Annotation of repetitive sequences***

TEs in the *E. ulmoides* genome were annotated by combining *de novo*- and homology-based approaches. For the *de novo* approaches, we used RepeatModeler (http://www.repeatma sker.org/RepeatModeler.html), RepeatScout (http://www.repeatmasker.org/) and LTR_FINDER to build the *de novo* repeat library^1^. For the homology-based approaches, we ran RepeatMasker (http://www.repeatmasker.org, version 3.3.0) against the Repbase TE library and RepeatProteinMask (http://www.repeatmasker.org/) against the TE protein database^3-5^.

The tRNA sequences in the genome were predicted using tRNAscan-SE^6^, and the predicted tRNA sequence was used to predict the primer binding sites (PBS) of LTR-RTs. A conserved domain (HMM) model of the pol gene (containing the AP, IN, RT, and RH domains), gag gene and env gene of LTR-RT was obtained from GyBD^7^, and was then used to predict presence and absence of the protein domains of the internal sequences of LTR-RTs. LTRharvest^6^ and LTRfinder were then used to identify full-length LTR-RTs based on structural features, and LTRdigest^8^ was used to annotate the LTR-RT structures. The original predicted results of LTRdigest were screened to accurately obtain the annotated LTR-RTs.

***Gene prediction***

Protein-coding gene structure predictions were conducted through a combination of homology, *de novo*, and transcriptome-based prediction methods. The protein sequences of nine plant genomes (*Arabidopsis thaliana*, *Camellia sinensis*, *Capsicum baccatum*, *Cuscuta australis*, *Hevea brasiliensis*, *Malus domestica*, *Oryza sativa*, *Salvia splendens*, and *T. kok-saghyz*) were obtained from Phytozome (https://phytozome.jgi.d oe.gov/pz/portal.html) and NCBI (https://www.ncbi.nlm.nih. gov/), and were then aligned to the *E. ulmoides* genome using Basic Local Alignment Search Tool (BLAST)^10^ and GeneWise^11^ to predict gene structures in the genome. *de novo* gene prediction on *E. ulmoides* genome was performed using Augustus^12^, GlimmerHMM^13^, and SNAP (http://homepage.mac.com / iankorf /). RNA-seq data derived from apical buds, young leaves, mature leaves, old leaves, bark, roots, peels, seeds, lateral buds, male flowers (SRX2447963, SRX2447934, SRX2447933, and SRX1160206), female flowers (SRX1160207), and stem tissue were assembled by Trinity^14^. These assembled sequences were aligned against the *E. ulmoides* genome by Program to Assemble Spliced Alignments (PASA)^15^. Valid transcript alignments were clustered based on genome mapping locations and then assembled into gene structures. In addition, RNA-seq reads were directly mapped to the genome using TopHat^16^ to identify putative exon regions and splice junctions. Cufflinks^17^ was then used to assemble the mapped reads into gene models. EVidenceModeler (EVM)^18^ was used to integrate the gene sets predicted by various methods into a non-redundant, more complete gene sets. Finally, PASA^15^ was combined with the transcriptome assembly results to correct the annotated results of EVM, and the information such as the UTR and variable shear were added to obtain the final gene set.

The final gene set obtained from gene structure annotation was compared with the Swiss-Prot^19^ and NR (https://www.ncbi.nlm.nih.gov/) protein databases by BLAST^20^ to annotate the protein-coding genes. The protein domains were obtained by searching Pfam^21^ and InterPro^22^, respectively. The gene ontology (GO)^23^ term for each gene was obtained from the corresponding InterPro or Pfam entry. The pathways in which genes might be involved were assigned using BLAST against the KEGG database^24^ with a cutoff E value < 1e−05.

Annotations for non-coding RNA include tRNA, rRNA, miRNA, and snRNA. TRNAscan-SE software^6^ was used to identify the tRNA sequences in the genome based on tRNA structural characteristics. Since rRNA species are highly conserved, the rRNA sequence of related species were used to identified rRNA sequence using BLAST alignment. Using the covariance model of INFERNAL software (http://infernal.janelia.org/) that comes with Rfam^21^, the miRNA and snRNA sequence information can be predicted from the genome.

The protein sequences from 12 species including *E. ulmoides，A. thaliana, D. carota, T. kok-saghyz, C. arabica, Catharanthus roseus, Actinidia chinensis, O. europaea, Camptotheca acuminata, Solanum tuberosum, H. brasiliensis* and *Dichanthelium oligosanthes* were used for gene family clustering analysis. When there are multiple transcripts in a gene, only the longest transcript in the coding region was selected for further analysis. In addition, genes encoding proteins with less than 50 amino acids were deleted. The filtered BLAST results were obtained between protein sequences of all species through BLASTP with a cutoff E value <1e-5. Using OrthoMCL^25^ with an expansion parameter equal to 1.5, the protein sequences from all 12 species were clustered into groups of paralogues and orthologues.

***Phylogenetic tree reconstruction***

MUSCLE^26^ was used to align the protein sequences from single-copy gene families from 12 species (*E. ulmoides，A. thaliana, D. carota, T. kok-saghyz, C. arabica, Catharanthus roseus, Actinidia chinensis, O. europaea, Camptotheca acuminata, Solanum tuberosum, H. brasiliensis* and *Dichanthelium oligosanthes*), and the alignment of each gene family was concatenated to the alignment matrix. A phylogenetic tree was constructed using RAxML^27^ with a maximum likelihood method and a bootstrap value of 100, where *D. oligosanthes* was designated as an outlier. Venn diagrams were constructed to show the number of gene families and specific gene families shared between five species (*E. ulmoides, O. europaea, S. tuberosum, C. arabica* and *C. roseus*) clustered into a group of phylogenetic trees.

***Species divergence time estimation***

The divergence time was estimated using MCMCtree in the PAML v4.0^28^. Parameters were as follows: a burn-in of 10,000 steps, a sample number of 100,000, a sample frequency of 2, and a clock of 2. The following calibration times of divergence were obtained from the TimeTree database^29,30^ and include: 110-124 Mya for *A. thaliana*, and *D. carota*, 97-109 Mya for *A. thaliana* and *H. brasiliensis*, 93-107 Mya for *D. carota* and *S. tuberosum*, and 77-91 Mya for *C. arabica* and *S. tuberosum*.

***Expansion and contraction of gene families***

All homologues in the 12 species identified by OrthoMCL^25^ were clustered, and CAFE^31^ was used to compare the cluster size differences between different species to determine the expansion and contraction of gene families^32^.

***Genome synteny and WGD***

Protein BLAST (BLASTP) was used to perform a homology search within the *E. ulmoides* genome and other related species genomes with a stringency of cutoff E value <1e-5 . Then, MCscan (http://chibba.agtec.uga.edu/duplication/mcscan/) software was used to search internal and collinear regions between *E. ulmoides* and other related species genomes. Sequence alignment of the homologous gene pairs in intra-genomic collinear regions or of inter-genomic collinear regions was performed, and the 4DTv and Ks values were calculated to identify the putative WGD events in the *E. ulmoides* genome and other related species. The molecular clock rate (r) was calculated to be 8.25×10^-9^ substitutions per synonymous site per year. The duplication time was estimated using the formula ks/2r ^33^ The collinearity analyses of *E. ulmoides* and *V. vinifera*, *and E. ulmoides* and *C. arabica* were conducted by JCVI^34^ (--cscore=.99), and the collinearity relationships between *E. ulmoides* and *V. vinifera* and between *E. ulmoides* and *C.canephora*  were tested using QUOTA­ALIGN^35^, and the collinearity dot plot was drawn.

***Transcriptome sequencing and analysis***

Different tissues, including mature leaves, the edges of peels, the centers of peels (the part covering the seeds), the xylem, and the seeds, of the 10-year-old diploid *E. ulmoides* trees were used for extracting RNA for performing RNA-seq experiment. Three biological replicates were used for each tissue. A total of 3 µg RNA per sample was used as input material for the RNA sample preparations. Sequencing libraries were generated using the NEBNext® Ultra™ Directional RNA Library Prep Kit for Illumina® (NEB, USA) following the manufacturer’s recommendations, and indexing sequences were added to attribute sequences to each sample. The clustering of the index-coded samples was performed on a cBot Cluster Generation System using the TruSeq PE Cluster Kit v3-cBot-HS (Illumina) according to the manufacturer’s instructions. After the cluster generation, the library preparations were sequenced on an Illumina HiSeq platform, and 125 bp/150 bp paired-end reads were generated. Raw data of fastq format were first processed through in-house Perl scripts. In this step, clean reads were obtained by removing reads containing adapter sequences, reads containing poly-N sequences and low-quality reads from the raw data. Additionally, the Q20, Q30 and GC content and the clean data were calculated. All downstream analyses were based on clean data with high quality. The index of the reference genome was built using Bowtie^36^, and paired-end clean reads were aligned to the reference genome using TopHat (http://ccb.jhu.edu/software/tophat/index.shtml). HTSeq v0.6.1^37^ was used to count the read numbers mapped to each gene. Then, the FPKM of each gene was calculated based on the length of the gene, and the read count mapped to this gene.

***Identification of resistance genes***

The RGAugury pipeline^38^ was used to screen the entire gene set used for RGA prediction. For BLASTP, the default cutoff E-value for initial RGA filtering was set to le−5.

***Identification of CGA synthesis-related genes and phylogenetic analysis***

To identify genes related to TPI and chlorogenic acid synthesis, the plant metabolic network (PMN v12.5) and KEGG databases were queried, and the *Arabidopsis* homologous genes were mined from the literature and downloaded. The corresponding gene family results were extracted and manually compared. HMMER and BLASTP were used as needed. MUSCLE (v3.8.31) was used for protein sequence alignment. The maximum likelihood phylogenetic tree was constructed using Mega-X (V10.0.5).

***Identification of transcription factors***

The identification and classification of TFs was performed by taking advanatage of annotated TF data stored in The Plant Transcription Factor Database^39^. The TF orthologs from three plants, *Arabidopsis thaliana*, *Solanum melongena* and *Eucalyptus grandis*, were identified through blast alignment with evalue >1e-5. The subject with highest bit score and identity was considered as the homologs of TFs from *E. ulmoides*. We manually searched existing literature extensively to identify paralogs that are evidenced to regulate secondary metabolites. Secondary metabolism related TFs of *E. ulmoides* was inferred using blast with tougher parameters evalue>1e-10 and identitiy >50%. GO annotation was designated using the Arabidopsis Information Resource (TAIR).

**References**

1. Li, P. *et al.* RGAugury: a pipeline for genome-wide prediction of resistance gene analogs (RGAs) in plants. *BMC Genom.* **17,** 852 (2016).
2. Osuna-Cruz, C. M. *et al.* PRGdb 3.0: a comprehensive platform for prediction and analysis of plant disease resistance genes. *Nucleic Acids Res.* **46,** D1197–D1201 (2017).
3. Xu, Z. & Wang, H. LTR_FINDER: an efficient tool for the prediction of full-length LTR retrotransposons. *Nucleic Acids Res.* **35,** W265–W268 (2007).

4. Chen, N. Using RepeatMasker to identify repetitive elements in genomic sequences. *Curr. Protoc. Bioinformatics* **Chapter 4,** Unit 4.10 (2004).

5. Price, A. L., Jones, N. C. & Pevzner, P. A. *De novo* identification of repeat families in large genomes. *Bioinformatics* **21,** i351– i358 (2005).

6. Lowe, T. M. & Eddy, S. R. tRNAscan-SE: a program for improved detection of transfer RNA genes in genomic sequence. *Nucleic Acids Res.* **25,** 955–964 (1997).

7. Llorens, C. *et al.* The Gypsy Database (GyDB) of mobile genetic elements: release 2.0. *Nucleic Acids Res.* **39,** D70–D74 (2011).

8. Ellinghaus, D., Kurtz, S. & Willhoeft, U. LTRharvest, an efficient and flexible software for *de novo* detection of LTR retrotransposons. *BMC Bioinform.* **9,** 18 (2008).

9. Steinbiss, S., Willhoeft, U., Gremme, G. & Kurtz, S. Fine-grained annotation and classification of *de novo* predicted LTR retrotransposons. *Nucleic Acids Res.* **37,** 7002–7013 (2009).

10. Kent, W. J. BLAT--the BLAST-like alignment tool. *Genome Res.* **12,** 656–664 (2002).

11. Birney, E., Clamp, M. & Durbin, R. GeneWise and genomewise. *Genome Res.* **14,** 988–995 (2004).

12. Keller, O., Kollmar, M., Stanke, M. & Waack, S. A novel hybrid gene prediction method employing protein multiple sequence alignments. *Bioinformatics* **27,** 757–763 (2011).

13. Majoros, W. H., Pertea, M. & Salzberg, S. L. TigrScan and GlimmerHMM: two open source ab initio eukaryotic gene-finders. *Bioinformatics* **20,** 2878–2879 (2004).

14. Grabherr, M. G. *et al.* Full-length transcriptome assembly from RNA-Seq data without a reference genome. *Nat. Biotechnol.* **29,** 644–652 (2011).

15. Haas, B. J. *et al.* Improving the Arabidopsis genome annotation using maximal transcript alignment assemblies. *Nucleic Acids Res.* **31,** 5654–5666 (2003).

16. Trapnell, C., Pachter, L. & Salzberg, S. L. TopHat: discovering splice junctions with RNA-Seq. *Bioinformatics* **25,** 1105–1111 (2009).

17. Kim, D. *et al.* TopHat2: accurate alignment of transcriptomes in the presence of insertions, deletions and gene fusions. *Genome Biol.* **14,** R36 (2013).

18. Haas, B. J. *et al.* Automated eukaryotic gene structure annotation using EVidenceModeler and the program to assemble spliced alignments. *Genome Biol.* **9,** R7 (2008).

19. Bairoch, A. & Apweiler, R. The SWISS-PROT protein sequence database and its supplement TrEMBL in 2000. *Nucleic Acids Res.* **28,** 45–48 (2000).

20. Altschul, S. F., Gish, W., Miller, W., Myers, E. W. & Lipman, D. J. Basic local alignment search tool. *J. Mol. Biol.* **215,** 403–410 (1990).

21. Griffiths-Jones, S. *et al.* Rfam: annotating non-coding RNAs in complete genomes. *Nucleic Acids Res.* **33,** D121–D124 (2005).

22. Hunter, S. *et al.* InterPro: the integrative protein signature database. *Nucleic Acids Res.* **37,** D211–D215 (2009).

23. Ashburner, M. *et al.* Gene ontology: tool for the unification of biology. The Gene Ontology Consortium. *Nat. Genet.* **25,** 25–29 (2000).

24. Ogata, H. *et al.* KEGG: kyoto encyclopedia of genes and genomes. *Nucleic Acids Res.* **27,** 29–34 (1999).

25. Li, L., Stoeckert, C. J., Jr. & Roos, D. S. OrthoMCL: identification of ortholog groups for eukaryotic genomes. *Genome Res.* **13,** 2178–2189 (2003).

26. Edgar, R. C. MUSCLE: multiple sequence alignment with high accuracy and high throughput. *Nucleic Acids Res.* **32,** 1792–1797 (2004).

27. Stamatakis, A. RAxML-VI-HPC: maximum likelihood-based phylogenetic analyses with thousands of taxa and mixed models. *Bioinformatics* **22,** 2688–2690 (2006).

28. Yang, Z. PAML 4: phylogenetic analysis by maximum likelihood. *Mol. Biol. Evol.* **24,** 1586–1591 (2007).

29. Hedges, S. B., Dudley, J. & Kumar, S. TimeTree: a public knowledge-base of divergence times among organisms. *Bioinformatics* **22,** 2971–2972 (2006).

30. Kumar, S., Stecher, G., Suleski, M. & Hedges, S. B. TimeTree: a resource for timelines, timetrees, and divergence times. *Mol. Biol. Evol.* **34,** 1812–1819 (2017).

31. De Bie, T., Cristianini, N., Demuth, J. P. & Hahn, M. W. CAFE: a computational tool for the study of gene family evolution. *Bioinformatics* **22,** 1269–1271 (2006).

32. Han, M. V., Thomas, G. W., Lugo-Martinez, J. & Hahn, M. W. Estimating gene gain and loss rates in the presence of error in genome assembly and annotation using CAFE 3. *Mol. Biol. Evol.* **30,** 1987–1997 (2013).

33. Moniz de Sa, M. & Drouin, G. Phylogeny and substitution rates of angiosperm actin genes. *Mol. Biol. Evol.* **13,** 1198–1212 (1996).

34. Tang, H., Krishnakumar, V. & Li, J. jcvi: JCVI utility libraries (Version v0.5.7). Zenodo (2015).

35. Tang, H. *et al.* Screening synteny blocks in pairwise genome comparisons through integer programming. *BMC Bioinform.* **12,** 102 (2011).

36. Langmead, B. & Salzberg, S. L. Fast gapped-read alignment with Bowtie 2. *Nat. Methods* **9,** 357–359 (2012).

37. Anders, S., Pyl, P. T. & Huber, W. HTSeq--a Python framework to work with high-throughput sequencing data. *Bioinformatics* **31,** 166–169 (2014).

38. Li, P. *et al.* RGAugury: a pipeline for genome-wide prediction of resistance gene analogs (RGAs) in plants. *BMC Genom.* **17,** 852 (2016).

39. Jin J.P. *et al*. PlantTFDB 4.0: toward a central hub for transcription factors and regulatory interactions in plants. *Nucleic Acids Research.* **45**:1040-1045 (2017).
