# Supplementary material for: High-quality de novo assembly of the Eucommia ulmoides haploid genome provides new insights into evolution and rubber biosynthesis
Source: Hortic Res. 2020 Nov 1;7:183. doi: 10.1038/s41438-020-00406-w (PMC7603500; doi:10.1038/s41438-020-00406-w)
Supplement: Supplementary file 3 — Figure S1-S19 [file 41438_2020_406_MOESM3_ESM.doc]

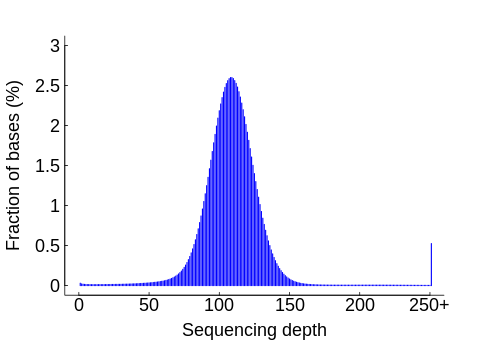


**Figure S1.** Sequencing depth distribution of the assembled genome.


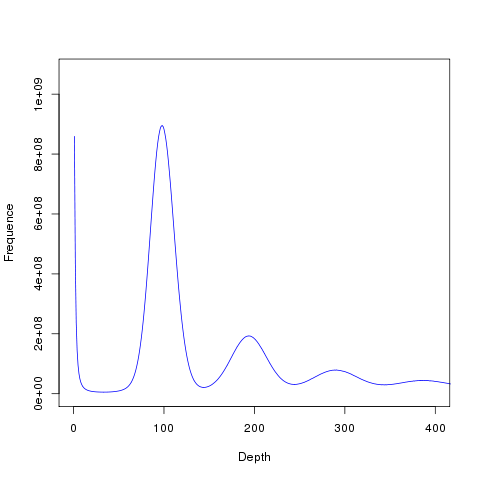


**Figure S2.** 17-mer analysis to estimate the *E. ulmoides* genome size. The x-axis and y-axis indicate the 17-mer number and frequency, respectively. The genome size and the ratio of repeat content are estimated to be 1.02 Gb and 65.90%, respectively. The heterozygosis simulation revealed that the heterozygosis ratio of *E. ulmoides* haploid was approximately 0.08%.


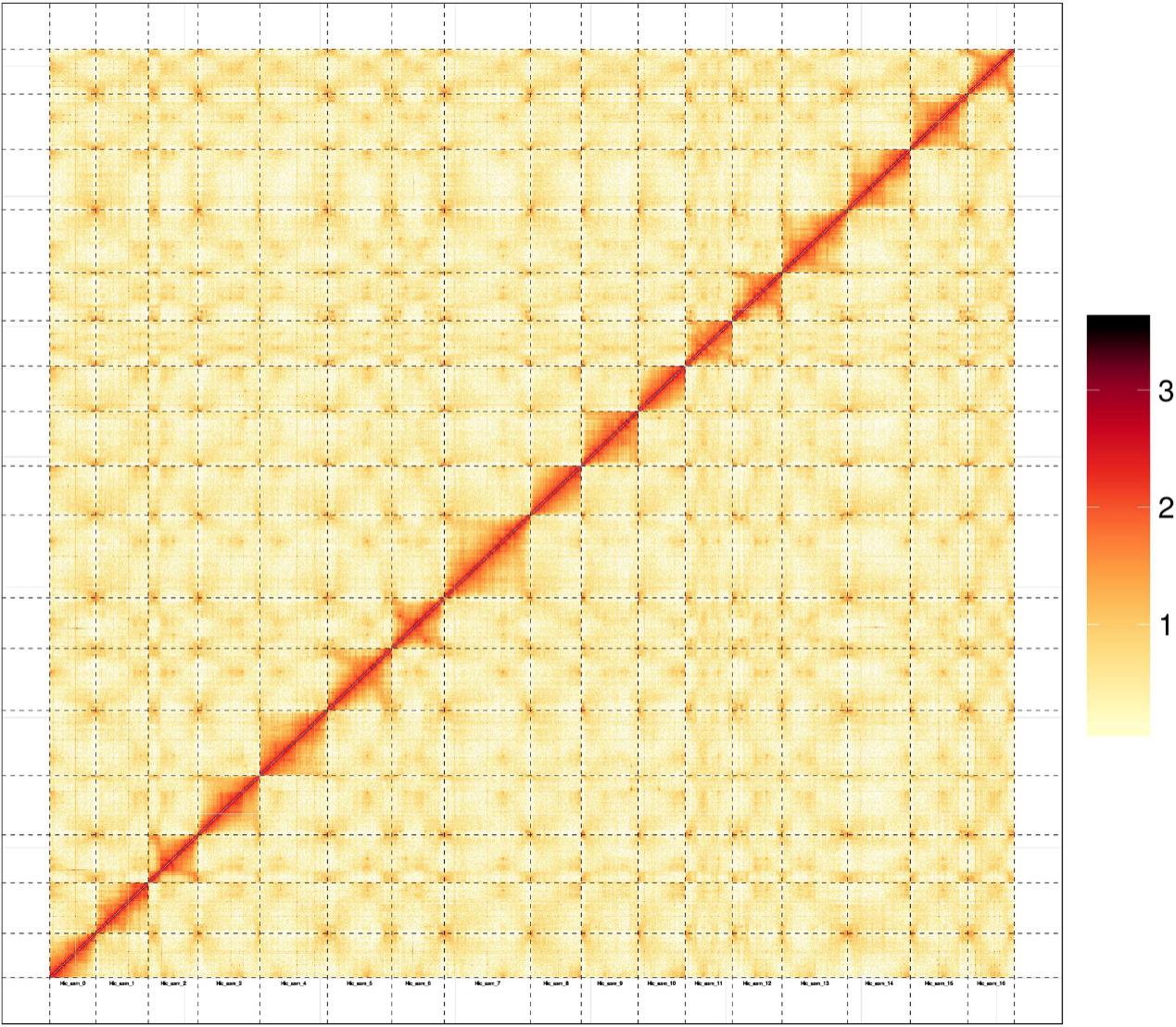


**Figure S3.** Heatmap showing Hi-C interactions in *Eucommia ulmoides* genome under a resolution of 500 kb. The color bar illuminates the degree of the Hi-C contact in the plot.


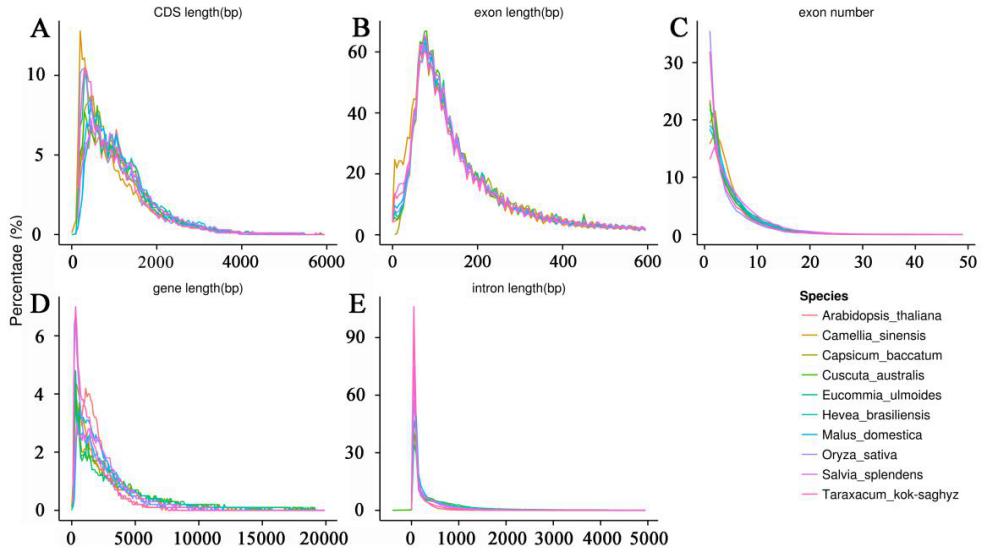


**Figure S4.** Cross-species comparisons of CDS length distribution (A), gene length

distribution (B), exon length distribution (C), gene length distribution (D) and intron length distribution (E).


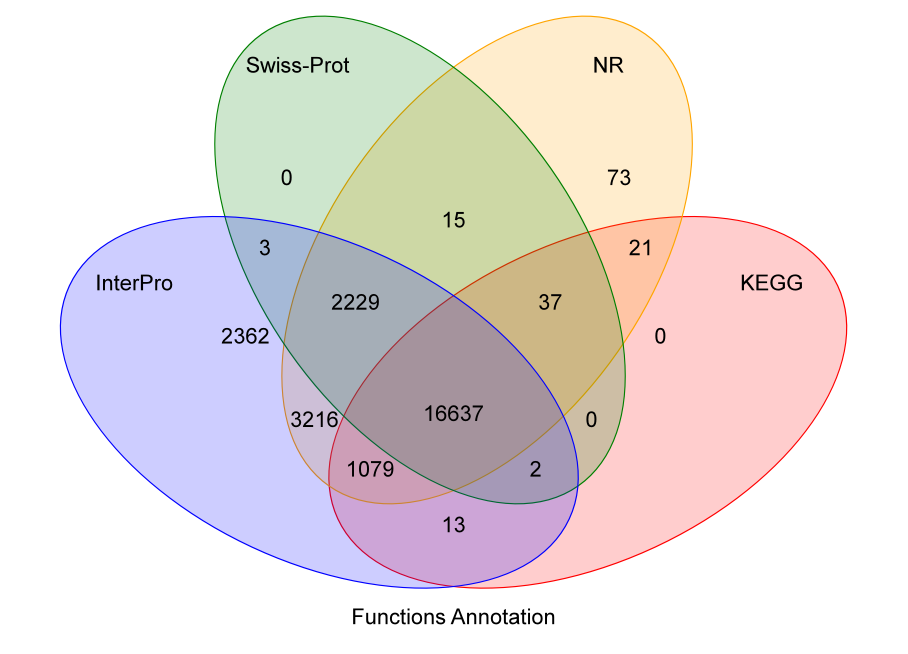


Figure S5. The functional annotations of gene models by InterPro, SwissProt, TrEMBL, GO and KEGG Pathway databases.


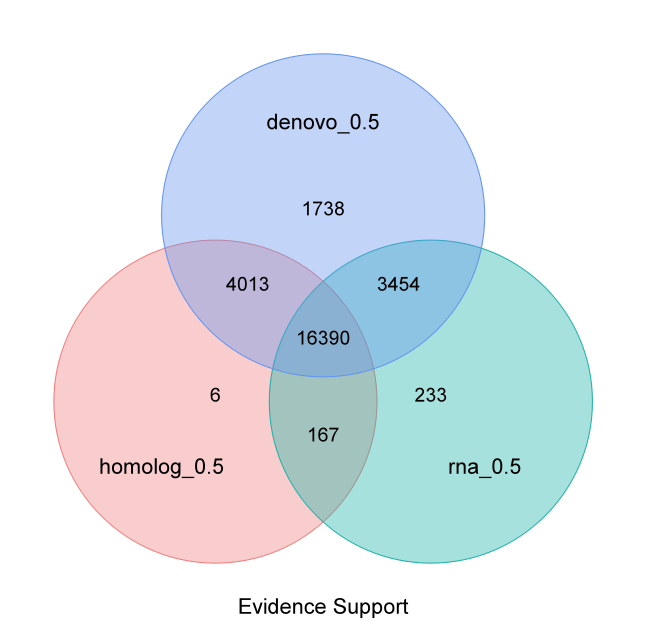


**Figure S6.** Summary of the genes predicted by three types of algorithms. *Ab initio*, homology-based, and transcript-based predictions were carried out for genome annotation. A total 26,001 genes were predicted in the *E. ulmoides* genome, and 16,390 predicted genes were shared among the three methods.


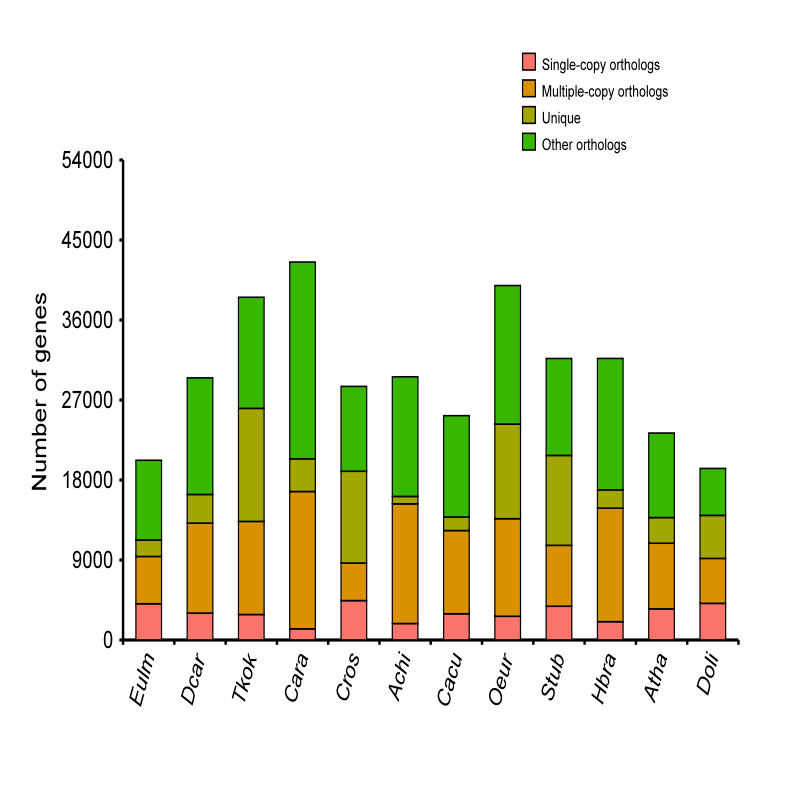


**Figure S7.** Clusters of orthologous and paralogous gene families in *E. ulmoides* and eleven more fully sequenced plant genomes. Only the longest isoform for each gene was used. Gene families were identified using the OrthoMCL package with the default parameters.


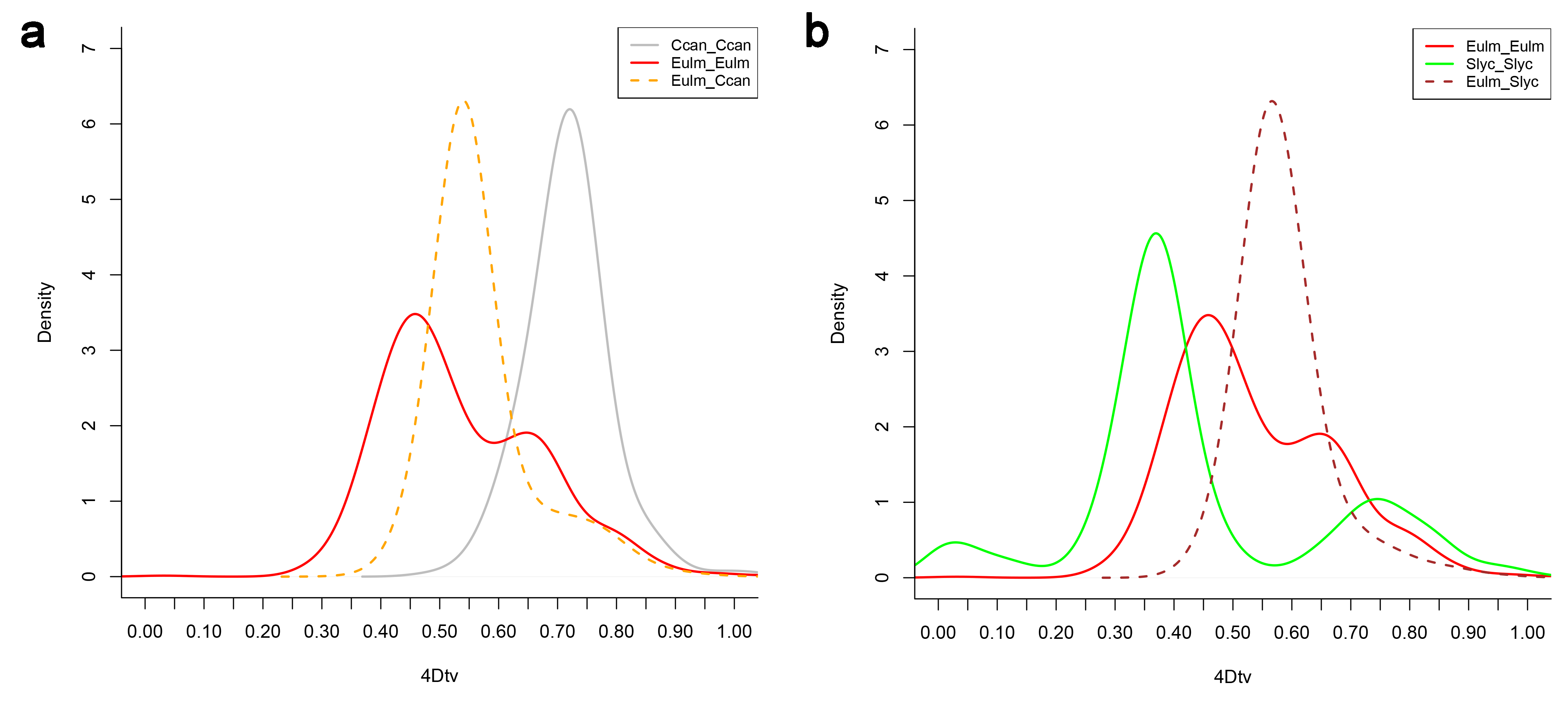


**Figure S8.** Density distributions of 4DTv for paralogous genes. The peak values are shown in insets.(a)Density distributions of fourfold-degenerate sites for paralogous genes. The peak values are shown in insets for *E. ulmoides*, and *C. canephora*. (b)Density distributions of fourfold-degenerate sites for paralogous genes. The peak values are shown in insets for *E. ulmoides*, and *S. lycopersicum*.


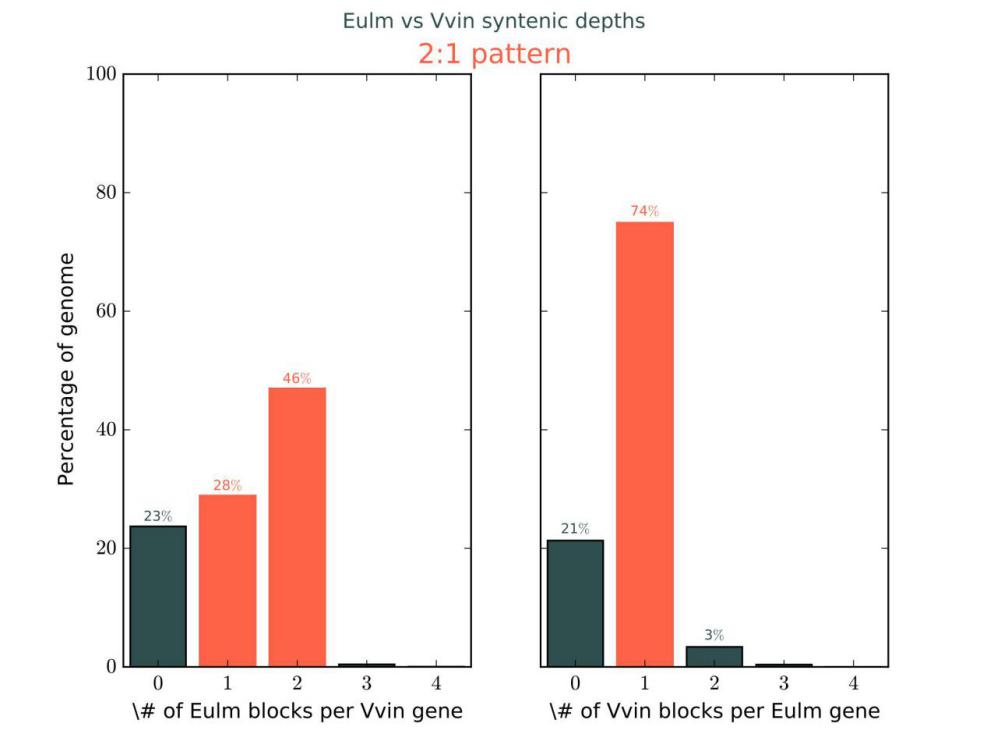


**Figure S9.** synteny pattern of *E. ulmoides* versus *V. vinifera.*


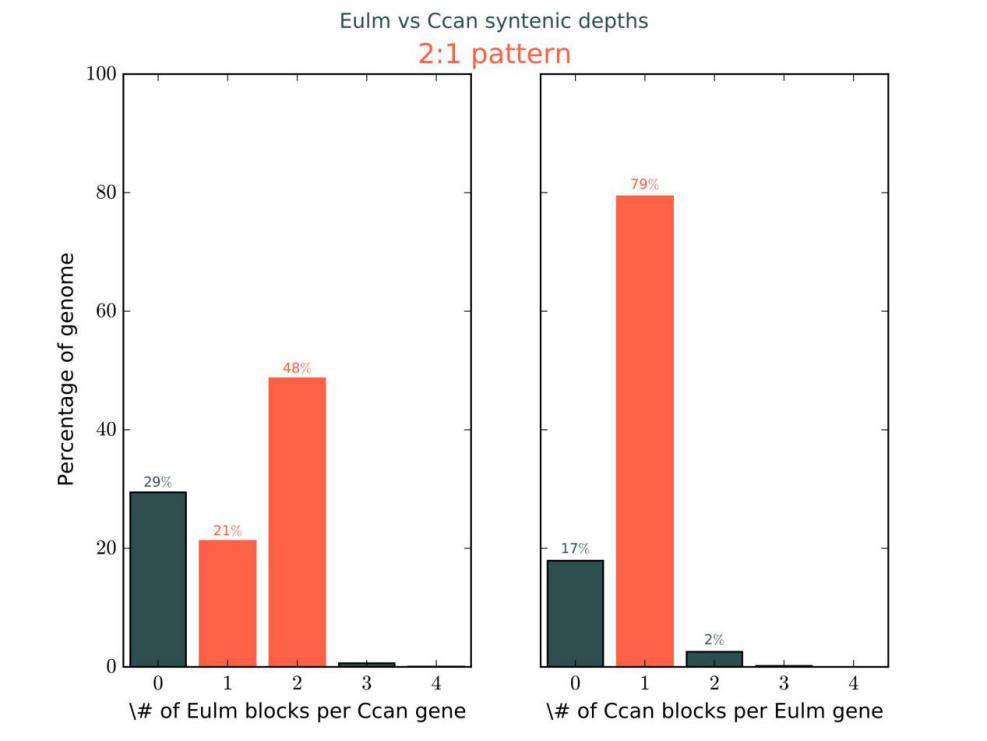


**Figure S10.** synteny pattern of *E. ulmoides* versus *C. arabica.*


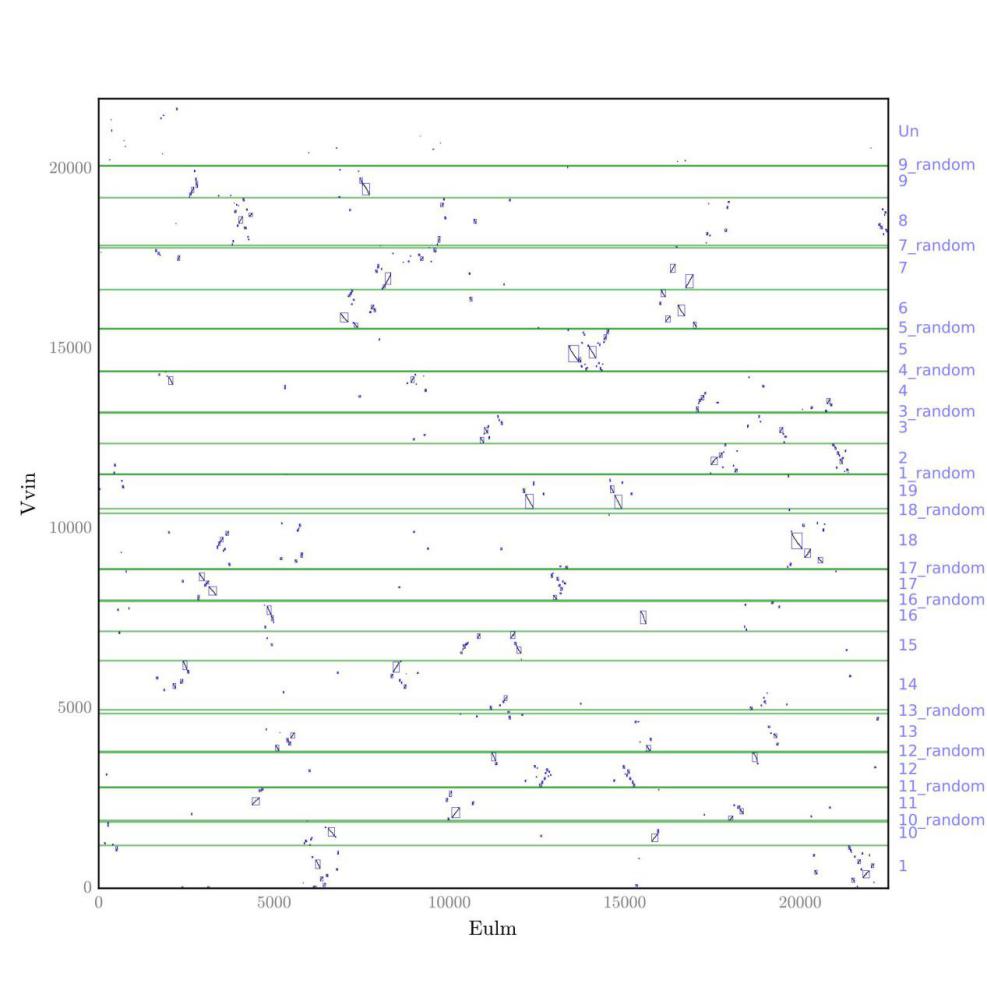


**Figure S11.** Syntenic dot plot of *E. ulmoides* versus *V. vinifera* (x-axis: *E. ulmoides* chromosomes; y-axis: *V. vinifera* chromosomes).


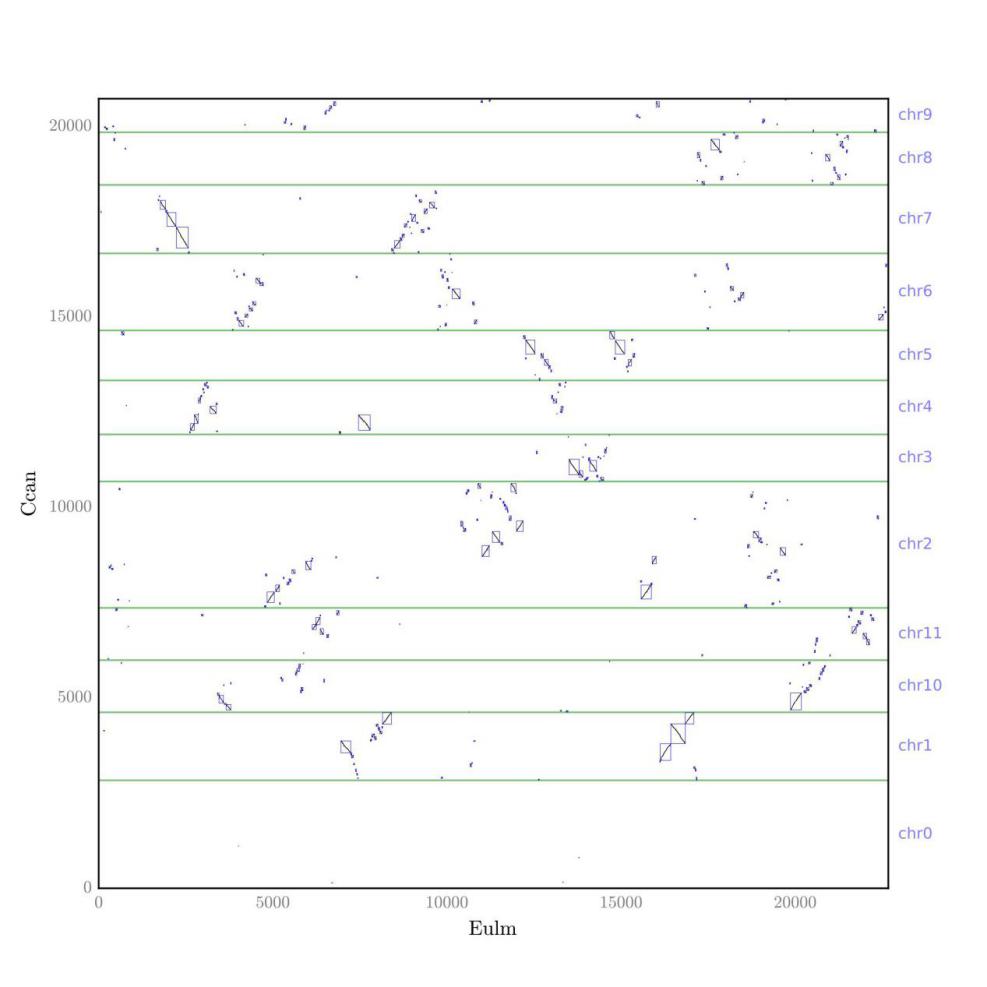


**Figure S12.** Syntenic dot plot of *E. ulmoides* versus *C. canephora* (x-axis: *E. ulmoides* chromosomes; y-axis: *C. canephora* chromosomes).


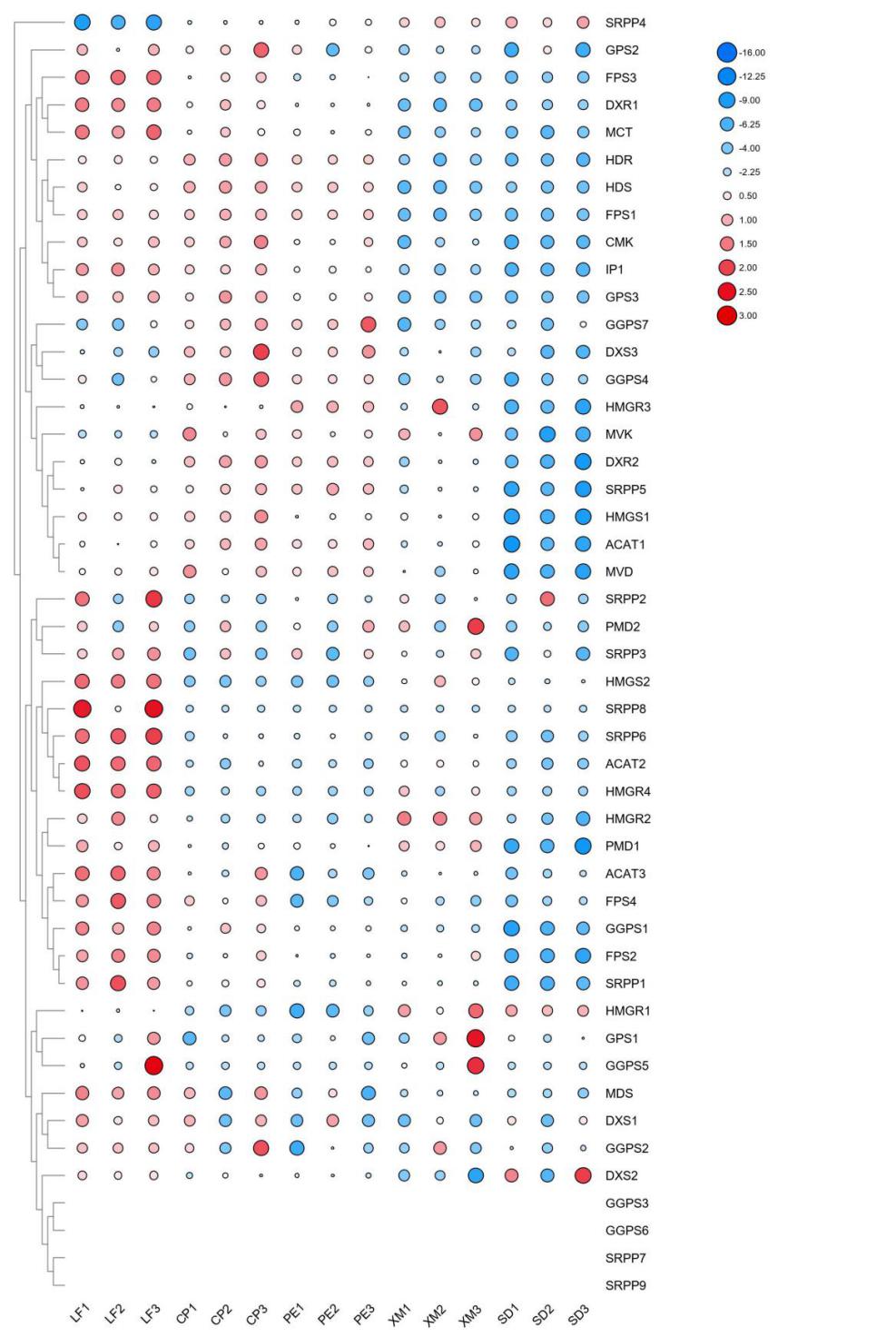


**Figure S13.** *E. ulmoides* rubber-related gene expression in selected tissues and the expression profiles of genes involved in the pathway. The expression level is presented by log2-transformed fragments mapped per kilobase of transcript length per million total mapped reads (log2-FPKM). LF1-3, leaf; CP1-3, central peel; PE1-3, peel edge; XM1-3, xylem; SD1-3, seed.


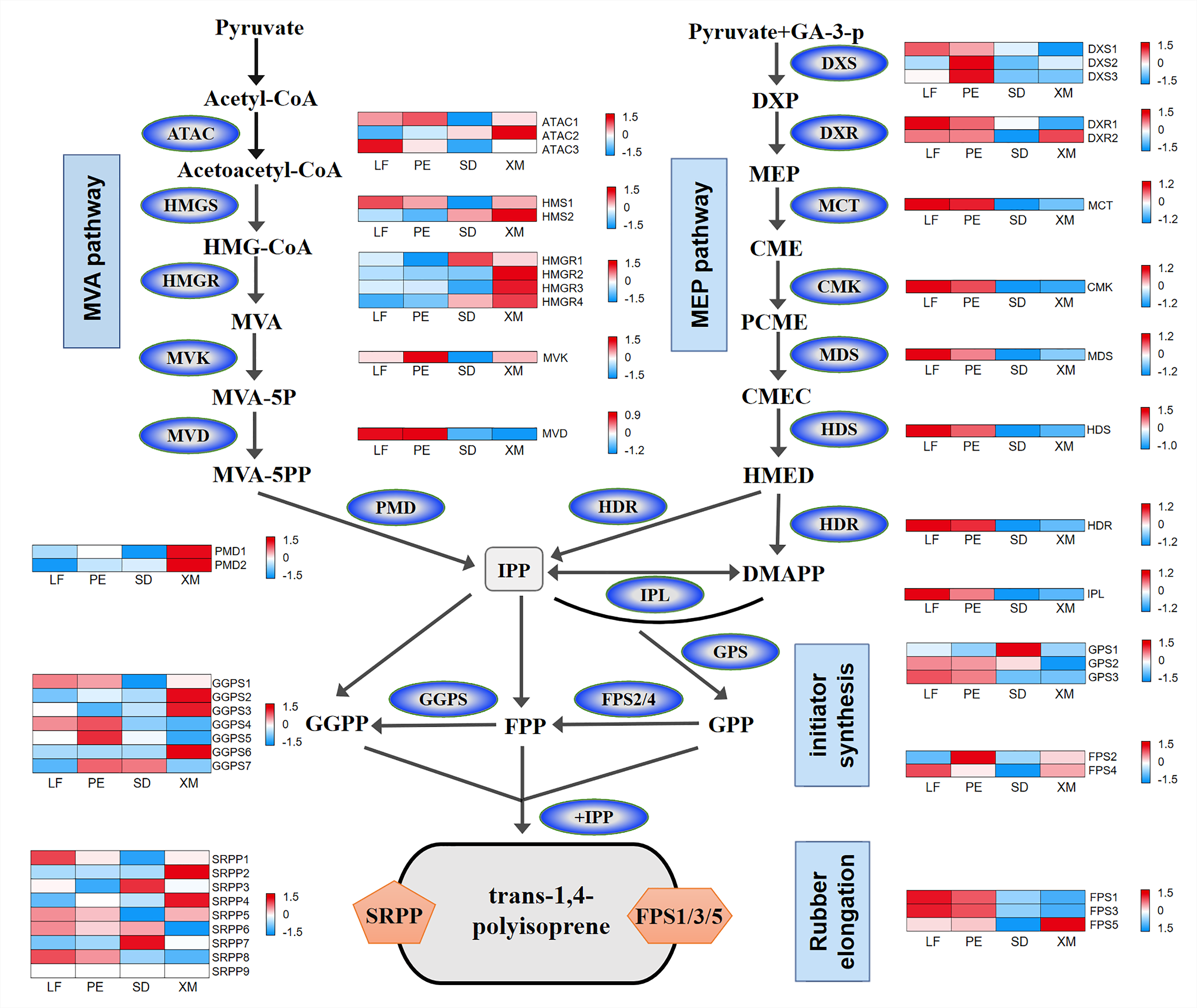


**Figure S14**. The *E. ulmoides* rubber biosynthesis pathway and expression profiles of genes involved in the pathway. The expression level is presented by log2-transformed fragments mapped per kilobase of transcript length per million total mapped reads (log2-FPKM). ACAT, acetyl-coenzyme A (CoA) C-acetyltransferase; HMGS, hydroxymethylglutaryl-CoA synthase; HMGR, hydroxymethylglutaryl-CoA reductase; MVK, mevalonate kinase; PMK, 5-phosphomevalonate kinase; MPD, mevalonate pyrophosphate decarboxylase; DXS, 1-deoxy-d-xylulose 5-phosphate synthase; DXR, 1-deoxy-d-xylulose 5-phosphate reductoisomerase; MCT, 2-C-methyl-d-erythritol 4-phosphate cytidylyltransferase; CMK, 4-(cytidine 5′-diphospho)-2-C-methyl-d-erythritol kinase; MDS, 2-C-methyl-d-erythritol 2,4-cyclodiphosphate synthase; HDS, 4-hydroxy-3-methylbut-2-enyl diphosphate synthase; HDR, 4-hydroxy-3-methylbut-2-enyl diphosphate reductase; IDI, isopentenyl diphosphate isomerase; GPS, geranyl diphosphate synthase; FPS, farnesyl diphosphate synthase; GGPS, geranylgeranyl diphosphate synthase; SRPP, small rubber particle protein; Acetyl-CoA, acetyl coenzyme-A; Acetoacetyl-CoA, 3-acetoacetyl-CoA; HMG-CoA, 3-hydroxy-3-methylglutaryl-CoA; MVA, mevalonate; MVA-5P, mevalonate-5-phosphate; MVA-5PP, mevalonate-5-diphosphate; GA-3-P, glyceraldehyde 3-phosphate; DXP, 1-deoxy-d-xylulose 5-phosphate; MEP, 2-C-methyl-d-erythritol 4-phosphate; CME, 4-(cytidine 5′-diphospho)-2-C-methyl-d-erythritol; PCME, 2-phospho-4-(cytidine 5′-diphospho)-2-C-methyl-d-erythritol; CMEC, 2-C-methyl-d-erythritol 2,4-cyclodiphosphate; HMED, 4-hydroxy-3-methylbut-2-enyl diphosphate; IPP, isopentenyl diphosphate; DMAPP, dimethylallyl diphosphate; GPP, geranyl diphosphate; FPP, farnesyl diphosphate; GGPP, geranylgeranyl diphosphate. LF, Leaf; PE, Peel; XM, Xylem; SD, Seed.


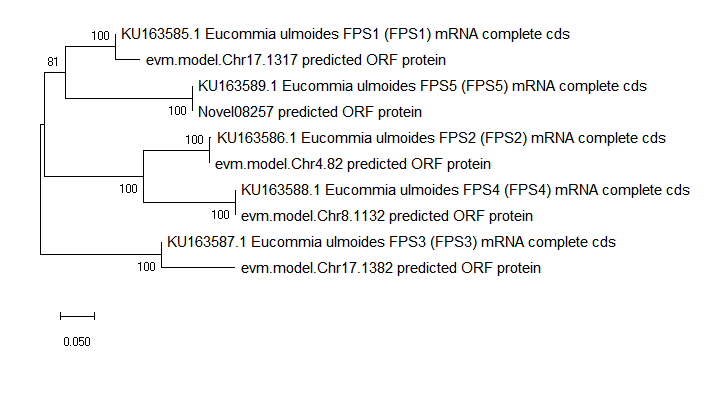


**Figure S15.** Phylogenetic analysis of FPSs from *E. ulmoides*v1.0 (Eu) and v2.0. Bootstrap values are shown at the nodes. Maximum likelihood (ML) tree for each SRPP genes was constructed using MEGA X with 1000 bootstrap replicates (WAG+I).


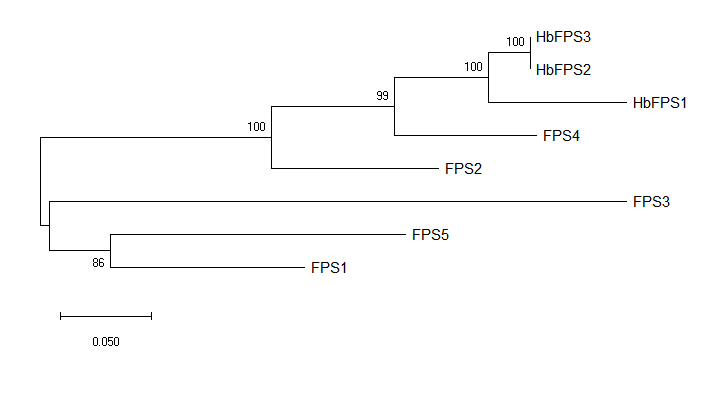


**Figure S16.** Phylogenetic analysis of FPSs from *E. ulmoides*(Eu) and *H. brasiliensis* (Hb). Bootstrap values are shown at the nodes. Maximum likelihood (ML) tree for each FPS genes was constructed using MEGA X with 1000 bootstrap replicates (WAG+I).


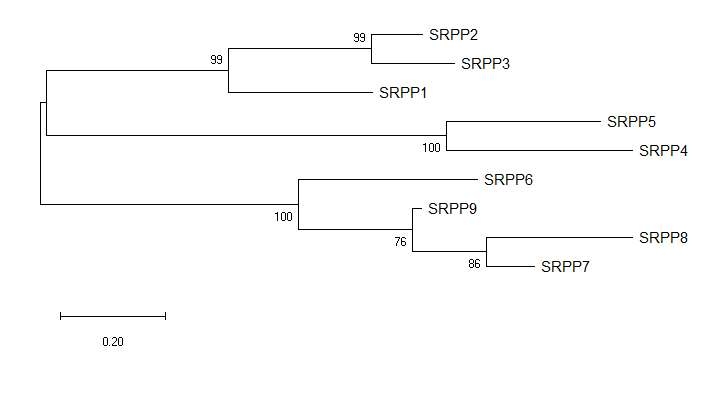


**Figure S17.** Phylogenetic analysis of SRPPs from *E. ulmoides* v2.0. Bootstrap values are shown at the nodes. Maximum likelihood (ML) tree for each SRPP genes was constructed using MEGA X with 1000 bootstrap replicates (JTT+G).


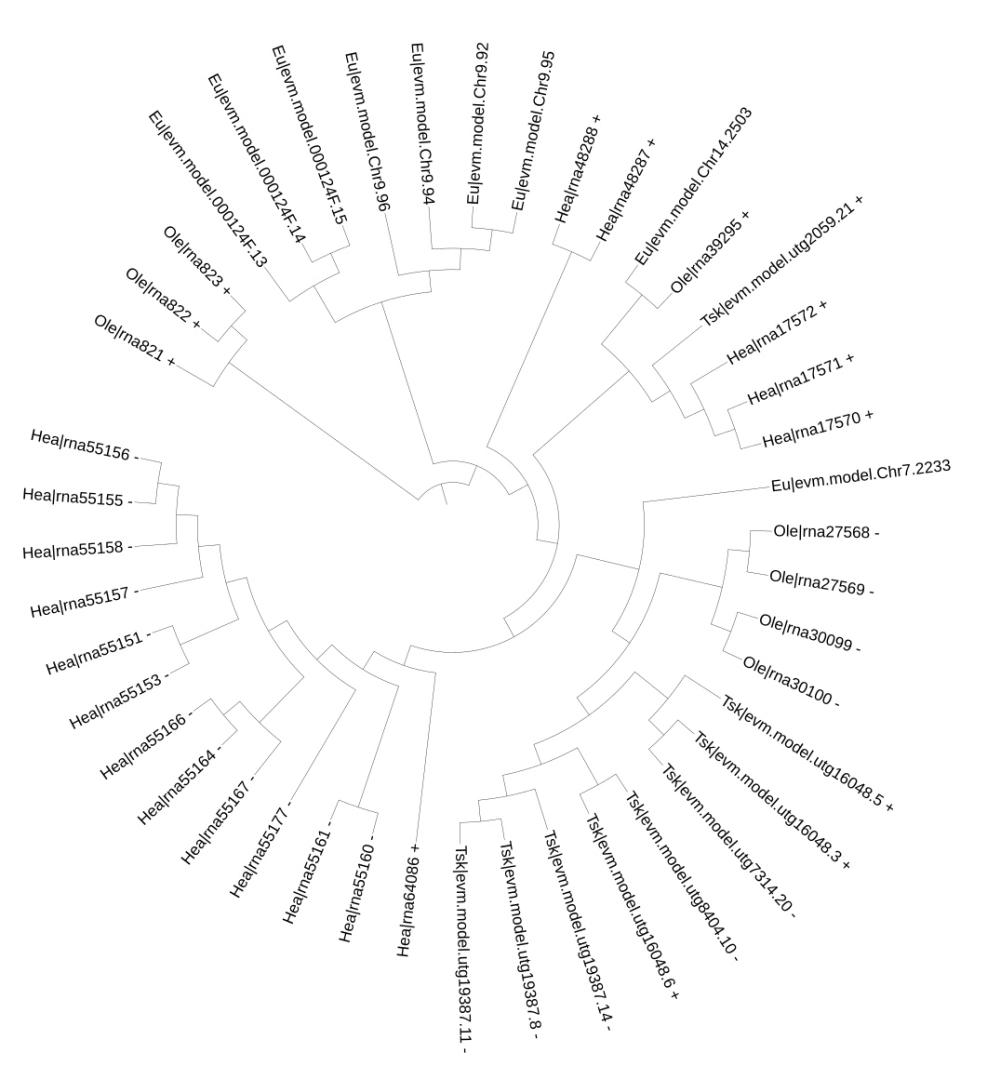


**Figure S18.** Phylogenetic tree of the REF/SRPP genes among *O. europaea*, *H. brasiliensis*, *T. kok-saghyz*, *E. ulmoides.* Maximum likelihood (ML) tree for each REF/SRPP gene was constructed using MEGA X with 1000 bootstrap replicates.


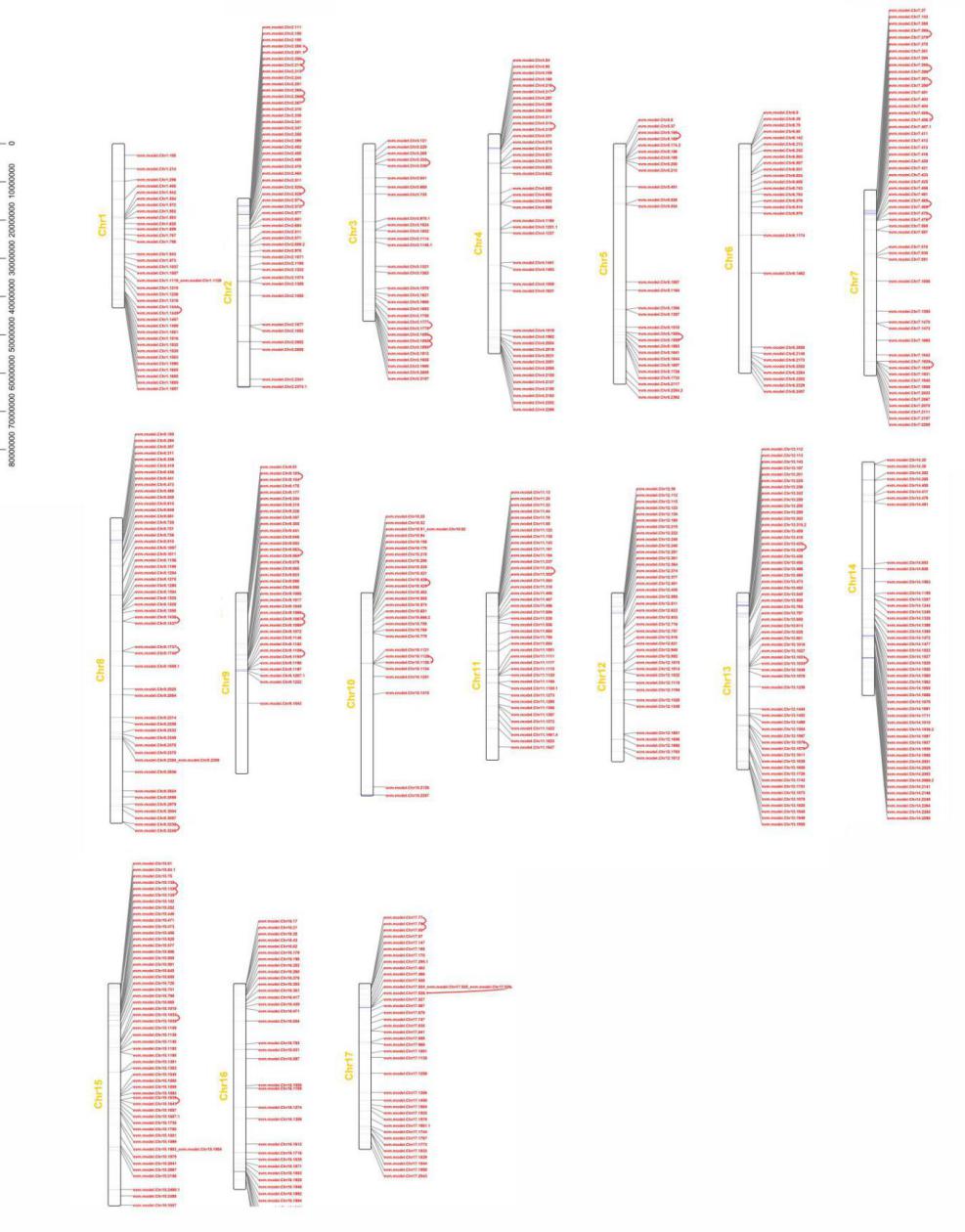


**Figure S19.** Distribution of RGAs gene on *Eucommia* chromosome.
