# Supplementary material for: High-quality de novo assembly of the Eucommia ulmoides haploid genome provides new insights into evolution and rubber biosynthesis
Source: Hortic Res. 2020 Nov 1;7:183. doi: 10.1038/s41438-020-00406-w (PMC7603500; doi:10.1038/s41438-020-00406-w)
Supplement: Supplementary file 4 — Supplemental table [file 41438_2020_406_MOESM4_ESM.docx]

| **Table S1. Statistics of *E. ulmoides* genome sequencing data** | | | | |
| --- | --- | --- | --- | --- |
| Pair-end libraries | Insert size | Total data(Gb) | Read length(bp) | Sequence coverage(X) |
| Illumina reads | 350bp | 142.05 | 150 | 138.75 |
| Pacbio | 40Kb | 97.26 | - | 95 |
| Hi-C reads | - | 133.74 | - | 130.63 |
| Total | - | 373.05 | - | 364.38 |

| **Table S2. Metrics of the different steps performed for *E. ulmoides* genome assembly** | | |
| --- | --- | --- |
|  | Contigs (PacBio) | Scaffolds (Hi-C corrected) |
| Number of sequences | 564 | 501 |
| Total length (Mb) | 947.84 | 947.86 |
| N50 (Mb) | 13.16 | 53.15 |
| N90 (Mb) | 2.91 | 43.43 |
| Longest | 34.99Mb | 79.92Mb |
| GC content | 35.17% | 35.17% |

| **Table S3. Statistical summary of final *E. ulmoides* genome assembly** | | | | |
| --- | --- | --- | --- | --- |
| Sample ID | Contig(bp) | Contig number | Scaffold length(bp) | Scaffold number |
| Total | 947,844,863 | 629 | 947,857,663 | 501 |
| Max | 30,532,491 | - | 79,924,447 | - |
| Number>=2000 | - | 609 | - | 481 |
| N50 | 11,154,905 | 27 | 53,150,112 | 8 |
| N60 | 7,865,838 | 37 | 48,634,971 | 10 |
| N70 | 5,505,488 | 52 | 46,496,334 | 12 |
| N80 | 3,723,523 | 74 | 44,264,498 | 14 |
| N90 | 1,869,606 | 107 | 43,436,084 | 16 |

| **Table S4. Statistics of each chromosome feature after clustering** | | | |
| --- | --- | --- | --- |
| Simplified_ID | Scffold number | Length（bp） | Gap numbr |
| Chr1 | 8 | 43033863 | 7 |
| Chr2 | 4 | 49442233 | 3 |
| Chr3 | 7 | 46496334 | 6 |
| Chr4 | 9 | 57468285 | 8 |
| Chr5 | 11 | 62926612 | 10 |
| Chr6 | 11 | 60118490 | 10 |
| Chr7 | 11 | 48634971 | 10 |
| Chr8 | 13 | 79924447 | 12 |
| Chr9 | 7 | 47534663 | 6 |
| Chr10 | 10 | 53150112 | 9 |
| Chr11 | 7 | 43807554 | 6 |
| Chr12 | 6 | 44264498 | 5 |
| Chr13 | 8 | 46397534 | 7 |
| Chr14 | 9 | 61157491 | 8 |
| Chr15 | 8 | 58177526 | 7 |
| Chr16 | 6 | 53926125 | 5 |
| Chr17 | 10 | 43436084 | 9 |
| Total | 145 | 899896822 | 128 |

| **Table S5. CEGMA evaluation results for the two *E. ulmoides* genome** | | | | |
| --- | --- | --- | --- | --- |
|  | complete | | complete+partial | |
|  | #Prots | %Completeness | #Prots | %Completeness |
| v1.0 | 196 | 79.03 | 232 | 93.55 |
| v2.0 | 230 | 92.74 | 238 | 95.97 |
| (1) #Prots represent number of 248 ultra-conserved CEGs present in genome; | | | |  |
| (2) %Completeness represent percentage of 248 ultra-conserved CEGs. | | | | |

| **Table S6. BUSCO evaluation results for two *E. ulmoides* genome** | |
| --- | --- |
|  | BUSCO notation assenssment results |
| v1.0 | C: 90.0%[S:86.0%,D:4.0%],F:2.8%,M:7.2%,n:1440 |
| v2.0 | C: 91.3%[S:87.9%,D:3.4%],F:1.7%,M:7.0%,n:1440 |
| (1) Species：Species name； | |
| (2) BUSCO notation assessment results：BUSCO evaluation results | |
| C：Complete Single-Copy BUSCOs | |
| D：Complete Duplicated BUSCOs | |
| F：Fragmented BUSCOs | |
| M：Missing BUSCOs | |
| n：Total BUSCO groups searched | |

| **Table S7. Small fragment library (350bp) reads coverage results for *E. ulmoides* genome** | | |
| --- | --- | --- |
|  |  | Percentage |
| Reads | Mapping rate | 99.18 |
| Genome | Average sequencing depth | 112.18 |
|  | Coverage (%) | 99.86 |
|  | Coverage at least 4X (%) | 99.79 |
|  | Coverage at least 10X (%) 99.71 | 99.71 |
|  | Coverage at least 20X (%) | 99.58 |

| **Table S8. Predicted protein-coding genes in the *E. ulmoides* genome** | | | | | | | |
| --- | --- | --- | --- | --- | --- | --- | --- |
|  | Gene set | Number | Average genes  length(bp) | Average CDS  length(bp) | Average exons  per gene | Average exon  length(bp) | Average intron  length(bp) |
| De novo | Augustus | 41734 | 3897.63 | 855.17 | 3.69 | 231.98 | 1132.54 |
|  | GlimmerHMM | 75705 | 11168.34 | 507.71 | 3.05 | 166.64 | 5208.57 |
|  | SNAP | 35590 | 5553.27 | 500.76 | 3.02 | 165.69 | 2498.56 |
|  | Geneid | 82611 | 4178.54 | 521.68 | 3.18 | 164.15 | 1678.96 |
|  | Genscan | 50840 | 11522.36 | 877.15 | 4.99 | 175.62 | 2664.97 |
| Homolog | Arabidopsis_thaliana | 40028 | 2761.25 | 846.38 | 2.83 | 299.56 | 1049.01 |
|  | Camellia_sinensis | 17442 | 5260.65 | 1196.7 | 3.58 | 333.92 | 1572.84 |
|  | Capsicum_baccatum | 24423 | 3573.19 | 1157.24 | 3.41 | 339.51 | 1003.07 |
|  | Cuscuta_australis | 35172 | 3013.6 | 1041.03 | 3.11 | 335.13 | 936.48 |
|  | Hevea_brasiliensis | 24867 | 3919.29 | 1216.36 | 3.68 | 330.56 | 1008.65 |
|  | Malus_domestica | 19688 | 3829.5 | 1325.95 | 3.75 | 353.61 | 910.47 |
|  | Oryza_sativa | 32409 | 2906.37 | 1076.59 | 2.84 | 378.85 | 993.48 |
|  | Salvia_splendens | 26386 | 3955.1 | 1158.05 | 3.65 | 317.19 | 1055.1 |
|  | Taraxacum_kok-saghyz | 49007 | 2425.91 | 870.53 | 2.69 | 323.96 | 921.88 |
| RNAseq | PASA | 78084 | 6087.5 | 1007.94 | 4.89 | 206.23 | 1306.64 |
|  | Cufflinks | 62153 | 9981.9 | 2372.8 | 6.21 | 382.13 | 1460.66 |
| EVM |  | 42078 | 4513.67 | 852.5 | 3.8 | 224.11 | 1305.68 |
| Pasa-update* |  | 41795 | 4575.28 | 859.9 | 3.81 | 225.9 | 1323.84 |
| Final set* |  | 26001 | 6138.21 | 1108.8 | 4.85 | 228.56 | 1305.93 |
| Note: * contains UTR area, others do not. | | |  |  |  |  |  |

| **Table S9. Statistical results of genetic structure of related species** | | | | | | |
| --- | --- | --- | --- | --- | --- | --- |
| **Species** | **Number** | **Average gene length(bp)** | **Average CDS length(bp)** | **Average exons per gene** | **Average exon length(bp)** | **Average intron length(bp)** |
| *Eucommia ulmoides* | 26,001 | 6,138 | 1,109 | 5 | 229 | 1305.93 |
| *Camellia sinensis* | 36,951 | 3,290 | 990 | 5 | 211 | 623.41 |
| *Salvia splendens* | 47,823 | 2,844 | 1,283 | 6 | 211 | 307.31 |
| *Oryza sativa* | 34,227 | 2,205 | 1,003 | 4 | 262 | 425.13 |
| *Taraxacum kok-saghyz* | 46,724 | 1,851 | 1,039 | 4 | 244 | 249.46 |
| *Malus domestica* | 47,821 | 4,776 | 1,210 | 5 | 248 | 920.02 |
| *Hevea brasiliensis* | 35,183 | 4,133 | 1,320 | 5 | 247 | 646.42 |
| *Arabidopsis thaliana* | 26,869 | 1,894 | 1,230 | 5 | 238 | 158.80 |
| *Capsicum baccatum* | 35,061 | 4,577 | 1,127 | 5 | 246 | 964.75 |
| *Cuscuta australis* | 19,341 | 3,992 | 1,207 | 5 | 228 | 648.13 |

| **Table S10. Summary of the functional annotation in *E. ulmoides* genome** | | | | | | | | |
| --- | --- | --- | --- | --- | --- | --- | --- | --- |
| Annotation database | | | Total gene number*(26001) | | | Annotated percent(%) | | |
| NR | | | 23307 | | | 89.6 | | |
| GO | | | 23304 | | | 89.6 | | |
| Pfam | | | 18286 | | | 70.3 | | |
| InterPro | | | 25541 | | | 98.2 | | |
| Swissprot | | | 18923 | | | 72.8 | | |
| KEGG Pathway | | | 17789 | | | 68.4 | | |
| Annotated | | | 25687 | | | 98.8 | | |
| Unannotated | | | 314 | | | 1.2 | | |
| * Number of genes that have been annotated in a corresponding database | | | | | |  | | |
| **Table S11. Non-coding genes in the *E. ulmoides* genome** | | | | | | | |  |
|  | Type | Copy number | | Average length (bp) | Total length (bp) | | Percentage of genome (%) |  |
| miRNA |  | 1032 | | 125.7 | 129720 | | 0.013686 |  |
| tRNA |  | 825 | | 75.05 | 61918 | | 0.006532 |  |
| rRNA | rRNA | 2099 | | 368.31 | 773073 | | 0.08156 |  |
|  | 18S | 362 | | 1463.24 | 529693 | | 0.055883 |  |
|  | 28S | 1181 | | 141.35 | 166934 | | 0.017612 |  |
|  | 5.8S | 293 | | 158.19 | 46351 | | 0.00489 |  |
|  | 5S | 263 | | 114.43 | 30095 | | 0.003175 |  |
| snRNA | snRNA | 875 | | 116.42 | 101865 | | 0.010747 |  |
|  | CD-box | 662 | | 106.12 | 70250 | | 0.007411 |  |
|  | HACA-box | 52 | | 149.31 | 7764 | | 0.000819 |  |
|  | splicing | 158 | | 148.18 | 23413 | | 0.00247 |  |

| **Table S12. Statistics of transposable elements and other repeats in the *E. ulmoides* genome** | | |
| --- | --- | --- |
| Type | Repeat size(bp) | Percentage of genome(%) |
| Trf | 57,632,989 | 6.08 |
| Repeatmasker | 547,677,901 | 57.78 |
| Proteinmask | 155,829,561 | 16.44 |
| Total | 592,433,730 | 62.5 |

| **Table S13. Repeat elements in the *E. ulmoides*** | | | |
| --- | --- | --- | --- |
| Type | Total number | Total length(bp) | Percent(%) |
| DNA/Academ | 3 | 2531 | 0.000404746 |
| DNA/CMC | 44062 | 20467476 | 3.27306 |
| DNA/DNA | 1593 | 580084 | 0.0927643 |
| DNA/Dada | 3 | 908 | 0.000145203 |
| DNA/Ginger | 1016 | 725219 | 0.115974 |
| DNA/Helitron | 12073 | 7352354 | 1.17575 |
| DNA/Kolobok | 19 | 26897 | 0.00430124 |
| DNA/MULE | 4607 | 3812613 | 0.609695 |
| DNA/Maverick | 2516 | 901331 | 0.144137 |
| DNA/MuLE | 17261 | 14774859 | 2.36273 |
| DNA/P | 1238 | 334365 | 0.0534701 |
| DNA/PIF | 5478 | 2685139 | 0.429395 |
| DNA/PiggyBac | 48 | 59219 | 0.00947002 |
| DNA/Sola | 3058 | 2361236 | 0.377598 |
| DNA/TcMar | 1409 | 624758 | 0.0999083 |
| DNA/Zisupton | 3 | 1818 | 0.000290726 |
| DNA/hAT | 15074 | 10115141 | 1.61757 |
| LINE/CR1 | 159 | 96194 | 0.0153829 |
| LINE/DRE | 16 | 8577 | 0.00137159 |
| LINE/Dong-R4 | 1 | 324 | 5.18E-05 |
| LINE/I | 51 | 60220 | 0.0096301 |
| LINE/Jockey | 431 | 245561 | 0.039269 |
| LINE/L1 | 17830 | 12352401 | 1.97534 |
| LINE/L2 | 1893 | 1148231 | 0.18362 |
| LINE/LOA | 3 | 330 | 5.28E-05 |
| LINE/Penelope | 10 | 117290 | 0.0187565 |
| LINE/Proto2 | 11 | 4698 | 0.000751282 |
| LINE/R1 | 33 | 24867 | 0.00397661 |
| LINE/R2 | 1446 | 576401 | 0.0921753 |
| LINE/RTE | 19804 | 7640778 | 1.22188 |
| LINE/Rex-Babar | 4 | 1884 | 0.00030128 |
| LINE/Tad1 | 5 | 49734 | 0.00795323 |
| LINE/Zorro | 5 | 2025 | 0.000323828 |
| LTR/Caulimoviru | 1432 | 1044919 | 0.167099 |
| LTR/Caulimovirus | 923 | 1473678 | 0.235664 |
| LTR/Copia | 224553 | 179533315 | 28.7101 |
| LTR/DIRS | 708 | 264891 | 0.0423601 |
| LTR/Delta | 1 | 813 | 0.000130011 |
| LTR/ERV | 18 | 5898 | 0.00094318 |
| LTR/ERV1 | 1692 | 1204003 | 0.192538 |
| LTR/ERV4 | 33 | 13752 | 0.00219915 |
| LTR/ERVK | 110 | 116357 | 0.0186073 |
| LTR/ERVL | 107 | 43908 | 0.00702156 |
| LTR/Foamy | 114 | 47433 | 0.00758526 |
| LTR/Gypsy | 245970 | 267694084 | 42.8084 |
| LTR/LTR | 67361 | 52206196 | 8.34857 |
| LTR/Lenti | 2 | 150 | 2.40E-05 |
| LTR/Pao | 69 | 559381 | 0.0894536 |
| Other/DNA_virus | 1 | 448 | 7.16E-05 |
| SINE/SINE | 177 | 113557 | 0.0181595 |
| SINE/tRNA | 616 | 149730 | 0.0239441 |
| Satellite/Satellite | 2547 | 1093017 | 0.17479 |
| Simple_repeat/Simple_repeat | 5558 | 2792021 | 0.446487 |
| Unknown/Unknown | 34740 | 29818143 | 4.76838 |
| Total | 737895 | 625331157 | 100 |

| **Table S14. Number of intact LTR elements identified by LTR-finder** | | |
| --- | --- | --- |
| Superfamily | Family | Number |
| Ty3/Gypsy | tat | 1218 |
| Ty3/Gypsy | reina | 352 |
| Ty3/Gypsy | galadriel | 253 |
| Ty3/Gypsy | del | 1680 |
| Ty3/Gypsy | crm | 611 |
| Ty3/Gypsy | athila | 1140 |
| Ty1/Copia | tork | 944 |
| Ty1/Copia | sire | 1010 |
| Ty1/Copia | retrofit | 966 |
| Ty1/Copia | oryco | 41 |
| Ty1/Copia | copia | 19 |
| Caulimoviridae | caulimoviridae_dom2 | 1 |
| Caulimoviridae | cavemovirus | 1 |
| Caulimoviridae | caulimovirus | 23 |
| Caulimoviridae | badnavirus | 5 |
| Total |  | 8264 |

| **Table S15. Summary of gene ortholog analysis conducted on 12 sequenced genomes** | | | | | | |
| --- | --- | --- | --- | --- | --- | --- |
| **Species** | **Gene number** | **Genes in families** | **Unclustered genes** | **Family number** | **Unique families** | **Average genes per family** |
| *Arabidopsis thaliana* | 28,869 | 23,283 | 5,586 | 12,972 | 736 | 1.79 |
| *Actinidia chinensis* | 32,744 | 29,612 | 3,132 | 14,015 | 289 | 2.11 |
| *Camptotheca acuminata* | 31,800 | 25,239 | 6,561 | 14,476 | 507 | 1.74 |
| *Catharanthus_roseus* | 33,829 | 28,531 | 5,298 | 13,466 | 732 | 2.12 |
| *Coffea arabica* | 44,516 | 42,521 | 1,995 | 14,579 | 1,051 | 2.92 |
| *Daucus carota* | 32,270 | 29,488 | 2,782 | 13,774 | 729 | 2.14 |
| *Dichanthelium oligosanthes* | 25,849 | 19,302 | 6,547 | 10,974 | 1,214 | 1.76 |
| *Eucommia ulmoides* | 26,001 | 20,219 | 5,782 | 13,494 | 539 | 1.50 |
| *Hevea brasiliensis* | 34,648 | 31,676 | 2,972 | 14,270 | 604 | 2.22 |
| *Olea europaea* | 50,373 | 39,876 | 10,497 | 14,375 | 1,255 | 2.77 |
| *Solanum tuberosum* | 39,000 | 31,670 | 7,330 | 13,951 | 1,188 | 2.27 |
| *Taraxacum kok-saghyz* | 46,724 | 38,559 | 8,165 | 14,513 | 2,047 | 2.66 |

| **Table S18. Chromosome duplication test results for *E. ulmoides*** | | | | |
| --- | --- | --- | --- | --- |
| Ratio | Eulm:Vvin | | Eulm:Ccan | |
|  | Eulm coverage (%) | Vvi coverage (%) | Eulm coverage (%) | Ccan coverage (%) |
| 1:1 | 64.6 | 92.5 | 61.5 | 94 |
| 2:1 | 96.2 | 95.5 | 97 | 97 |
| 3:1 | 96.4 | 95.6 | 97.3 | 96.9 |
| 4:1 | 96.4 | 95.6 | 97.3 | 96.9 |
| 6:1 | 96.4 | 95.6 | 97.3 | 96.9 |
| 8:1 | 96.4 | 95.6 | 97.3 | 96.9 |
| Eulm: *Eucommia ulmoides*,Vvin: *Vitis vinifera*. Ccan: *Coffea canephora* | | | | |
| Ratio 1:1 means no WGD after γ event; 2:1 means a doubling after γ event; 3:1 means a tripling after γ event; 4:1 means two tripling after γ event; 6:1 means a tripling after γ event; 8:1 means three tripling after γ event. When ratio reaches the correct ratio, the eulm coverage will hardly increase. The analysis results are shown in the table below. When the ratio is 2:1, the eulm coverage will reach a high value, and the later ratio will not rise more than 0.5%. It is speculated that a doubling will occur after the γ event; the collinear lattice diagram is drawn with the ratio of 2:1, which also has a more obvious 2:1 relationship. | | | | |

| **Table S19. Estimating the occurrence time of WGD events based on KS peak** | | | | | | |
| --- | --- | --- | --- | --- | --- | --- |
|  |  | |  | Age |  | r |
| Ccan_Ccan |  | 2.0-2.2 |  |  | 121-133Mya | 8.25E-09 |
| Eulm_Eulm | 1.15 | 1.8-2.0 |  | 69.7Mya | 109.1-121.1 | 8.25E-09 |
| Slyc_Slyc | 0.8 | 2.0 |  | 48.5 | 121Mya | 8.25E-09 |

| **Table S23. List of CGA genes and their expression (Fragments per kilobase of  transcript per million fragments mapped, FPKM) in two *E. ulmoides* tissues** | | | | | | | | |
| --- | --- | --- | --- | --- | --- | --- | --- | --- |
| **Item** | **Gene name** | **Gene id** | **FPKM in two tissues** | | | | | |
|  |  |  | **Leaf** | | | **Bark** | | |
| 1 | PAL1 | evm.model.Chr7.1716 | 6.36 | 3.35 | 5.64 | 3.63 | 2.79 | 1.27 |
| 2 | PAL2 | evm.model.Chr11.579 | 240.44 | 208.80 | 217.89 | 51.88 | 62.96 | 69.26 |
| 3 | PAL3 | evm.model.Chr5.974 | 19.81 | 15.74 | 25.09 | 0.93 | 0.47 | 1.57 |
| 4 | PAL4 | evm.model.Chr9.1656 | 30.27 | 32.11 | 26.16 | 0.00 | 0.00 | 0.00 |
| 5 | PAL5 | evm.model.Chr7.1720 | 166.75 | 202.05 | 188.38 | 175.79 | 117.73 | 78.94 |
| 6 | PAL6 | evm.model.Chr17.1344 | 0.26 | 0.00 | 2.58 | 0.27 | 0.85 | 3.88 |
| 7 | PAL7 | evm.model.Chr9.1102 | 2.04 | 2.56 | 3.10 | 7.86 | 3.62 | 1.33 |
| 8 | C4H1 | evm.model.Chr14.1919 | 474.31 | 527.92 | 491.69 | 0.70 | 0.62 | 5.77 |
| 9 | C4H2 | evm.model.Chr16.590 | 0.42 | 0.35 | 0.00 | 0.00 | 0.00 | 0.00 |
| 10 | 4CH1 | evm.model.Chr10.185 | 39.32 | 45.53 | 45.80 | 60.63 | 69.83 | 60.33 |
| 11 | 4CH2 | evm.model.Chr15.787 | 0.00 | 0.00 | 0.00 | 0.00 | 0.00 | 0.00 |
| 12 | 4CH3 | evm.model.Chr2.1520 | 15.90 | 11.50 | 14.02 | 1.85 | 2.23 | 3.46 |
| 13 | 4CH4 | evm.model.Chr5.906 | 31.30 | 32.03 | 28.53 | 33.56 | 40.53 | 52.95 |
| 14 | 4CH5 | evm.model.Chr7.1445 | 0.03 | 0.00 | 0.00 | 0.00 | 0.00 | 0.00 |
| 15 | 4CH6 | evm.model.Chr7.8 | 127.88 | 139.94 | 130.29 | 0.00 | 0.00 | 0.00 |
| 16 | 4CH7 | evm.model.Chr8.84 | 17.95 | 17.32 | 13.89 | 15.11 | 12.43 | 17.83 |
| 17 | 4CH8 | evm.model.Chr9.122 | 1.75 | 0.69 | 2.48 | 0.00 | 0.00 | 0.00 |
| 18 | C3′H1 | evm.model.Chr4.1406 | 10.48 | 5.04 | 9.23 | 40.41 | 44.10 | 17.79 |
| 19 | C3′H2 | evm.model.Chr9.1941 | 132.60 | 138.76 | 143.37 | 28.36 | 25.39 | 18.24 |
| 20 | HCT | evm.model.Chr10.213 | 54.45 | 31.86 | 41.97 | 0.93 | 0.57 | 1.32 |
| 21 | HQT1 | evm.model.Chr11.1345 | 3.98 | 4.23 | 3.96 | 2.34 | 1.08 | 2.10 |
| 22 | HQT2 | evm.model.Chr16.430 | 91.01 | 63.58 | 88.12 | 24.98 | 23.52 | 21.76 |
| 23 | HQT3 | evm.model.Chr13.968 | 0.55 | 0.00 | 0.00 | 0.00 | 0.00 | 0.00 |

| **Table S26. The statistics of gaps in two *E. ulmoides* genome assembly** | | |
| --- | --- | --- |
|  | v1.0 | v2.0 |
| Total gaps number | 104,772 | 128 |
| Gaps number per Mb | 99.78 | 0.14 |

| **Table S27. Pest and disease resistance genes identified in the *E. ulmoides* genome** | | | | | | | | | | | | |
| --- | --- | --- | --- | --- | --- | --- | --- | --- | --- | --- | --- | --- |
| Spicies | NBS encoding | | | | | | | | RLP | RLK | TM-CC | Total |
|  | NBS | CNL | TNL | CN | TN | NL | TX | other |  |  |  |  |
| *Eucommia* | 24 | 17 | 19 | 9 | 10 | 29 | 19 | 12 | 73 | 441 | 108 | 761 |

| **Table S28. Overview of the gene duplication analysis of *E. ulmodes* RGAs gene** | | | | | | | | | |
| --- | --- | --- | --- | --- | --- | --- | --- | --- | --- |
|  | Class | R protein type | Singleton | Tandem | Proximal | WGD | Dispersed | Total no. genes | Ratio of dispersed R gene to total no. (%) |
| NBS Coding | CN | CC-NBS | 0 | 2 | 1 | 0 | 6 | 9 | 66.67% |
|  | CNL | CC-NBS-LRR | 0 | 5 | 9 | 0 | 3 | 17 | 17.65% |
|  | N | NBS | 0 | 4 | 5 | 2 | 13 | 24 | 54.17% |
|  | NL | NBS-LRR | 0 | 7 | 5 | 0 | 17 | 29 | 58.62% |
|  | TN | TIR-NBS | 0 | 2 | 2 | 0 | 6 | 10 | 60.00% |
|  | TNL | TIR-NBS-LRR | 0 | 5 | 8 | 2 | 4 | 19 | 21.05% |
|  | TX | TIR | 0 | 5 | 4 | 0 | 10 | 19 | 52.63% |
|  | Others | CC-TIR | 0 | 4 | 5 | 1 | 2 | 12 | 16.67% |
| RLK | | receptor like kinase | 0 | 63 | 63 | 101 | 214 | 441 | 48.53% |
| RLP | | receptor like protein | 1 | 13 | 12 | 5 | 42 | 73 | 57.53% |
| TM-CC | | transmembrane coiled-coil-containing | 3 | 7 | 3 | 23 | 72 | 108 | 66.67% |
| Total | |  | 4 | 117 | 117 | 134 | 389 | 761 | 51.12% |
